# Metabolite profiles of diabetes mellitus and response to intervention in anti-hyperglycemic drugs

**DOI:** 10.3389/fendo.2023.1237934

**Published:** 2023-10-31

**Authors:** Yanzhong Liu, Dan Wang, Yi-Ping Liu

**Affiliations:** Provincial University Key Laboratory of Sport and Health Science, School of Physical Education and Sport Sciences, Fujian Normal University, Fuzhou, China

**Keywords:** diabetes mellitus, metabolites, amino acids, lipids, carbohydrates

## Abstract

Type 2 diabetes mellitus (T2DM) has become a major health problem, threatening the quality of life of nearly 500 million patients worldwide. As a typical multifactorial metabolic disease, T2DM involves the changes and interactions of various metabolic pathways such as carbohydrates, amino acid, and lipids. It has been suggested that metabolites are not only the endpoints of upstream biochemical processes, but also play a critical role as regulators of disease progression. For example, excess free fatty acids can lead to reduced glucose utilization in skeletal muscle and induce insulin resistance; metabolism disorder of branched-chain amino acids contributes to the accumulation of toxic metabolic intermediates, and promotes the dysfunction of β-cell mitochondria, stress signal transduction, and apoptosis. In this paper, we discuss the role of metabolites in the pathogenesis of T2DM and their potential as biomarkers. Finally, we list the effects of anti-hyperglycemic drugs on serum/plasma metabolic profiles.

## 1 Introduction

According to the latest statistics from the International Diabetes Federation (IDF), as of 2021, about 537 million adults worldwide had diabetes, and this number is expected to increase to 783 million by 2045, causing 6.7 million deaths and high health costs every year (1). T2DM is the most common type of diabetes mellitus, accounting for about 90% of the total number of diabetes mellitus. It is of great significance to explore the pathogenesis of T2DM and develop precise and reliable prevention and treatment strategies. T2DM is a typical metabolic disease, usually accompanied by the disorder of systemic metabolic networks including carbohydrates, lipids, and amino acids, which is very suitable for metabolomics and lipidomics studies. Metabolites are not only ending products of genome regulation and cellular energy transfer, reflecting biological situations that have occurred or are occurring in the body, but also have multiple functions such as signaling molecules, immune regulation, and environmental sensors. Thus, the exploration of metabolite changes can reflect the metabolic phenotype of T2DM in a relatively comprehensive way. In contrast to biopsies, blood sample collection is a minimally invasive method with the advantages of rapid, economical and high availability, and is essential for facilitating mapping of disease metabolic profiles and prognostic diagnosis.

## 2 Metabolite profiles of T2DM

**Figure 1:**
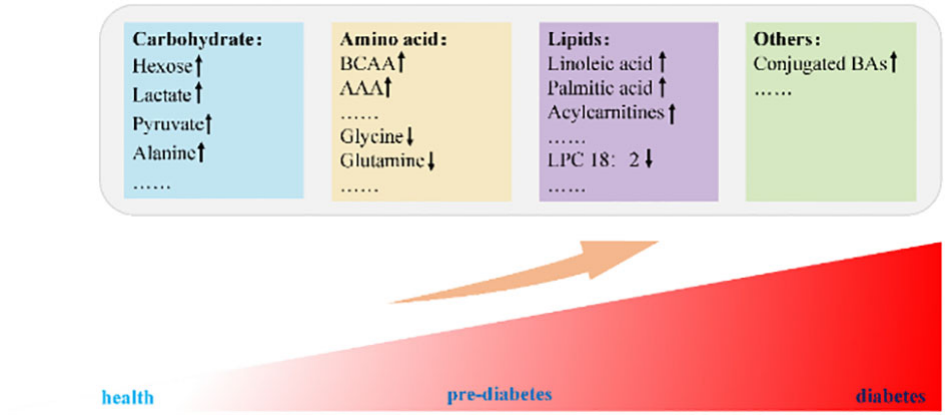
BCAA, branched-chain amino acids; AAA, aromatic amino acids; LPC, lysophosphatidylcholine; BAs, bile acids.

Metabolites are commonly recognized as end products of a wide range of gene transcription and biochemical reactions, and there is growing evidence that metabolites can be involved in disease development as biomarkers (**Figure 1**) and regulators. When acting as regulators, metabolites have an impact on the pathogenesis of T2DM in at least the following aspects: (1) Metabolites can regulate the downstream signaling pathway of insulin and directly affect insulin sensitivity; (2) Accumulation of harmful metabolic intermediates; (3) Cause organelle dysfunction; (4) Directly or indirectly mediates the inflammatory response of target tissues. A comprehensive understanding of metabolic pathways may be a novel direction for the prevention and treatment of T2DM.

### 2.1 Carbohydrate

Chronic hyperglycemia is not only a major feature of diabetes, but also a key factor in accelerating its progression and inducing complications. High glucose can damage the mitochondrial aerobic metabolic flux of pancreatic β-cells and reduce insulin content, which may be the pathological basis of the progressive decline of β-cell function in patients with T2DM. Excess fructose can cause a dramatic increase in hepatocyte carbohydrate response element binding protein (ChREBP) activity, which not only mediates changes in circulating triglycerides and high density lipoprotein (HDL) levels, but also is an important upstream regulator of a key enzyme in BCAA metabolism, branch chain ketoate dehydrogenase kinase (BCKDK)/metal ion-dependent protein phosphatase (PPM1K), integration affects BCAA oxidation and lipid metabolism (2). Evidence suggests that overproduction of the subtype ChREBP-β mediates glucose toxicity and subsequent cell death in β-cells. Overexpression of the different subtypes of ChREBP-α enhances glucose-stimulated β-cell proliferation and antagonizes Chrebp-β-cell death mediated by Nuclear factor erythroid 2-related factor 2 (Nrf2) antioxidant pathway (3).

Single-cell sequencing results showed that genes related to oxidative phosphorylation and ATP synthesis were significantly downregulated in the islets of T2DM patients (4), and this phenomenon was verified in animal models. Nearly all glycolytic enzymes were significantly upregulated in the diabetic mouse islets. In contrast, genes, proteins, and BCAA metabolic pathways associated with mitochondrial oxidative phosphorylation were significantly reduced (5, 6). Haythorne et al. have found that the impairment of β-cell function by high glucose is not glucose per se, but mediated by metabolic intermediates associated with increased glycolysis flux, one or more metabolites located between phosphofructokinase (PFK) and glyceraldehyde-3-phosphate dehydrogenase (GAPDH). By stimulating mammalian rapamycin complex 1 (mTORC1) and inhibiting AMPK activity, preventing pyruvate from entering the TCA cycle, and the imbalance of NADH/NAD^+^ in mitochondria and cytoplasm exacerbates the accumulation of upstream metabolites of GAPDH, creating a vicious cycle. This may partly explain the impaired oxidative phosphorylation of mitochondria. More importantly, glucose stimulation at 8mM appears to be sufficient to initiate this cycle, suggesting that cumulative impairment of pancreatic function may have already begun in patients with early impaired glucose tolerance (7).

**Figure 2:**
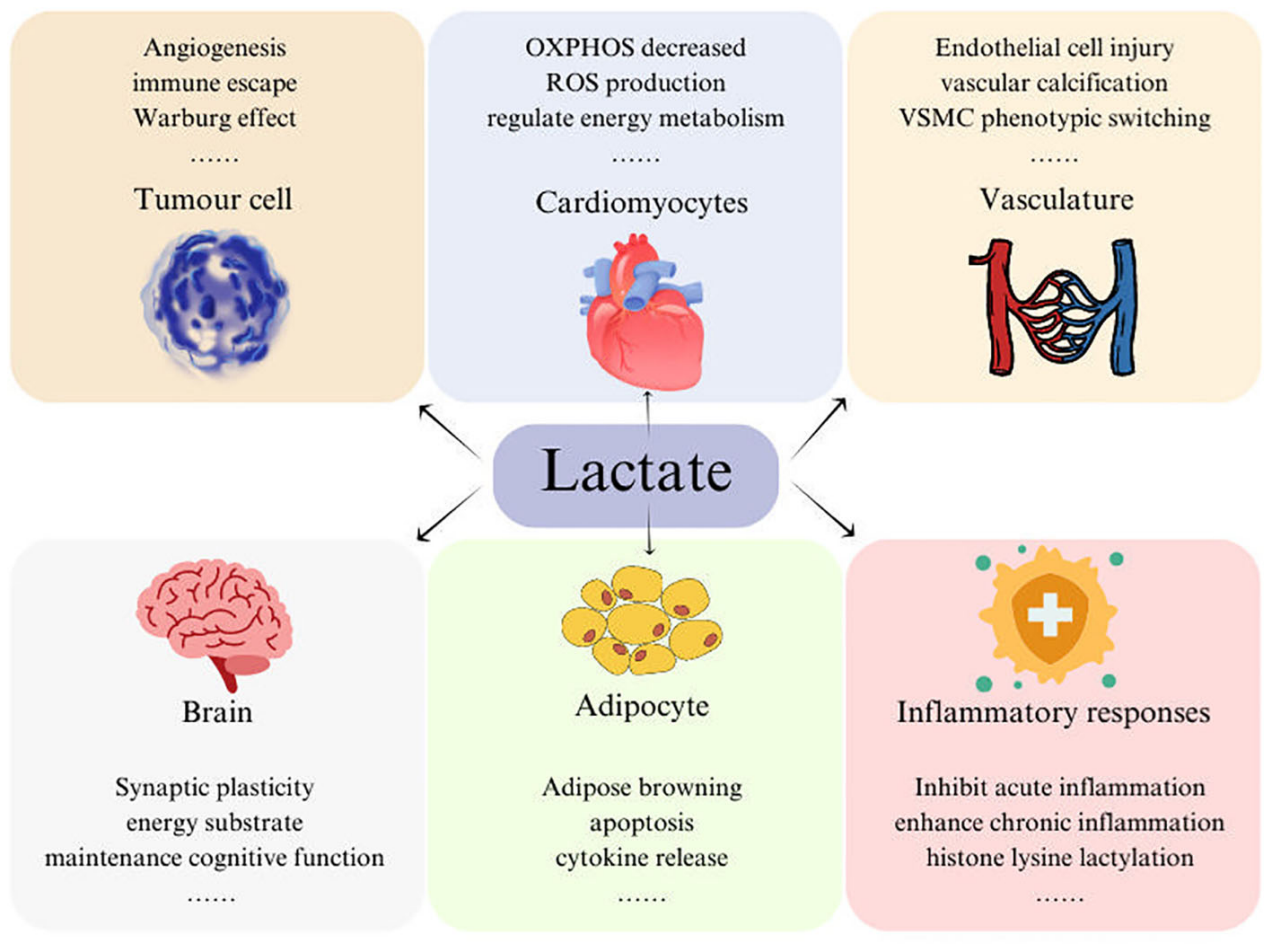
Lactate is involved in physiological and pathological processes of various tissues, including cardiovascular disease, immune response, fatty acid metabolism, cognitive function, and tumor cell angiogenesis. OXPHOS, oxidative phosphorylation; VSMC, vascular smooth muscle cell.

Impaired glucose oxidative phosphorylation metabolic pathways and increased glycolysis flux are determinants of increased lactate levels. In patients at high risk of CVD, plasma lactate, pyruvate, glycerol-3 phosphate, and isocitrate were significantly positively correlated with the risk of T2DM (23%-44% higher for every 1 SD increase) (8, 9), and was associated with various pathological phenomena of T2DM: (1) Increased blood lactate concentration reflects decreased mitochondrial oxidation capacity and is strongly positively correlated with IR index (10); (2) Blood lactate level may reflect liver dysfunction in T2DM patients (9); (3) Blood lactate levels may be indicative of susceptibility to T2DM to some extent (11). However, the effect of lactate as a signaling molecule on disease is complex and may depend on exposure duration and specificity of tissue and organ (**Figure 2**). Recent evidence suggests that lactate signaling is involved in inflammatory response (12–14), cells proliferation and migration (15), appetite regulation (16), redox homeostasis regulation (17), histone modification (18), and vascular cells damage (19–21). Diabetes is commonly accompanied by oxidation and systemic chronic inflammation. Elevated lactate levels can lead to an increase in NADH/NAD^+^ ratio, and mitochondria actively oxidize lactate to produce additional ROS accordingly. When antioxidants are out of balance, oxidative damage may be caused. Lactate is one of the main fuels of TCA cycle. Acute lactate exposure can stimulate mitochondrial coupling efficiency and promote bioenergetics of mitochondria in heart, skeletal muscle, and liver (22), while chronic hyper-lactate exposure may negatively affect mitochondrial respiration rate, reduced metabolic flexibility (23).

Chronic hyperglycemia can also trigger the activation of collateral glucose metabolic pathways, such as polyol, hexosamine, and AGE, resulting in oxidative stress, promoting the transcription of pro-inflammatory factors and apoptosis, and thus contribute to the occurrence and development of diabetes mellitus and its complications. AGE, cytokines and ROS can promote the production of triose phosphate, an intermediate of glucose metabolism, and increase *de novo* synthesis of diacylglycerol (DAG), the activator of protein kinase C (PKC) (24). In the polyol pathway, aldose reductase activated by hyperglycemia reduces glucose to sorbitol, which is further metabolized to fructose by sorbitol dehydrogenase. An increase in plasma fructose concentration is positively correlated with the development of T2DM and can lead to liver insulin resistance (IR) and non-alcoholic fatty liver disease development (25–28). In tissues such as nerves, sorbitol cannot easily cross the cell membrane, so the accumulation of sorbitol and fructose can increase the osmotic pressure in the cytoplasm and cause the leakage of myoinositol, a deficiency of which can lead to the production of DAG. On the other hand, the activation of the polyol pathway leads to the consumption of NADPH and promotes the conversion of NAD^+^ to NADH, which may not only reduce the production of the antioxidant glutathione, resulting in oxidative stress (29), but also competitively inhibit GAPDH, down-regulate glycolytic flux, and promote the transfer of more glucose to non-traditional metabolic pathways (such as hexosamine), thus aggravating glucotoxicity (30, 31). As the hexosamine biosynthesis pathway is also activated in diabetes, fructose-6-phosphate is detached from glycolysis and subsequently catalyzed to glucosamine 6-phosphate by fructose 6-phosphate aminotransferase. Glucosamine 6-phosphate forms the end product uridine diphosphate-N-acetylglucosamine through acetylation and isomerization. It then serves as the basic substrates for the formation of glycosyl side chains in post-translational modifications of proteins and lipids (32). This post-translational modification can aggravate glucotoxicity by regulating target protein stability, activity and subcellular localization, which can promote liver gluconeogenesis, lead to impaired insulin signaling and pancreatic β-cell function (33), and is directly involved in the pathogenesis of several diabetic complications, especially in cardiovascular disease and kidney dysfunction (34).

### 2.2 Amino acid

Since Felig et al. found in the 1970s that the increased concentration of circulating amino acids in obese people is associated with decreased insulin sensitivity (35), a large number of studies have confirmed the value of amino acids in the early identification and risk stratification of diabetes and its complications (36–40). Among the known and relatively clear amino acid biomarkers, plasma branched-chain amino acids (BCAA) and aromatic amino acids (AAA) increased significantly (**Table 1**), while glycine and glutamine decreased in diabetes and prediabetes (25, 41–43, 52–56).

**Table 1:** Association of amino acids profiles with T2DM in cohort studies.

Sample size (incident cases)	Duration of follow-up(years)	Platform	Metabolites	End point	OR/HR (95% CI)	Ref
769	6	NMR	BCAA, phenylalanine, alanine, tyrosine↑ Glutamine**↓**	HOMA-IR	2.09^a^(Men)	(36)
189	12	LC-MS	BCAA, tyrosine, phenylalanine**↑**	Diabetes	1.70-2.42^a^	(40)
91	2	LC-MS	Glycine**↓**	T2DM	0.85^a^	(41)
76	5	MS-MS	BCAA, phenylalanine, alanine, glutamine and glutamate**↑** Aspartate/asparagine, glycine**↓**	T2DM	1.56-2.22^a^ 0.42-0.58^a^	(42)
340	19	NMR	BCAA, alanine, isoleucine, phenylalanine, tyrosine**↑** Glycine**↓**	Diabetes	1.27-1.48^a^ 0.77^a^	(43)
9180	5.7	LC-MS	Tryptophan**↑**	T2DM	—	(44)
251	3.8	LC-MS	Tryptophan**↑**	T2DM	1.29^b^	(45)
17	1.5	LC-MS/MS	BCAA**↑**	HOMA-IR	—	(46)
70	5.5	MS-MS	Aspartic acid/asparagine, phenylalanine**↑** Histidine**↓**	Prediabetes	2.39-2.72^a^ 0.89-0.90^a^	(47)
540	—	NMR	isoleucine, alanine**↑**	Liver/muscle HOMA-IR	—	(48)
152	1-3	LC-MS	isoleucine, alanine, proline**↑** glycine, arginine**↓**	HOMA-IR	—	(49)
151	9.5	UHPLC-MS/MS	BCAA, alanine, glutamate, arginine**↑** glycine**↓**	T2DM	—	(50)
16	2.3	NMR	BCAA**↑**	IR	—	(51)

HOMA-IR, homeostasis model assessment for insulin resistance; IR, insulin resistance; NMR, nuclear magnetic resonance; LC-MS, liquid chromatography-mass spectrometry; MS-MS, tandem mass spectrometry; GC-MS, gas chromatography-mass spectrometry; IGT, impaired glucose tolerance; BCAA, branched-chain amino acids; T2DM, type 2 diabetes mellitus; SDMA, symmetric dimethylarginine; **↑**, increased; **↓**, decreased; —, not available, ^a^, odd ratio (OR), ^b^, hazard ratio (HR).

BCAA is most closely related to homeostasis model assessment for insulin resistance (HOMA-IR) and blood glucose (46, 57, 58). The results of large sample size genome-wide association studies (GWAS) show that BCAA contributes to the increase of the incidence of IR and T2DM (59). Under physiological conditions, BCAA promotes protein synthesis or inhibits its breakdown by activating the mTOR signaling pathway, a catalytic subunit of two distinct structural and functional complexes mTORC1 and mTORC2. mTORC1 promotes protein synthesis and regulates autophagy, and mTORC2 is a classic insulin/PI3K signaling pathway effector (60). BCAA supplementation alone did not significantly affect skeletal muscle mass and glycemic control in patients with T2DM (61), nor worsen diet-induced insulin resistance and glucose intolerance in obese mice (62). Whereas HFD combined with BCAA supplementation caused chronic activation of mTORC1, p70-S6 kinase (p70S6K), and phosphorylation of insulin receptor substrate 1 (IRS1) serine, promoting the accumulation of multiple acylcarnitines in muscle, decreased insulin sensitivity (37), which can be reversed by the mTOR inhibitor rapamycin (63). Reducing dietary BCAA intake rapidly reduced diet-induced obesity, improved glucose tolerance, reversed fatty acyl-coA accumulation in skeletal muscle, normalized glycine content, and improved skeletal muscle insulin sensitivity (64, 65). In leucine-incubated skeletal muscle, AMPK activity decreased by more than 50%, phosphorylation of mTOR and p70S6K was concentration-dependent, phosphorylation of insulin-stimulated Akt was impaired, and AMPK agonist was used to inhibit these changes (66).

Paradoxically, Leucine has also been suggested to increase GLUT4-mediated glucose uptake, stimulate insulin-dependent PI3K and protein kinase C (PKC) signaling cascades, and increase mitochondrial biogenesis and substrate oxidation capacity (67, 68). Leucine supplementation has been shown to reduce body weight by 32% and improve insulin sensitivity, plasma total cholesterol, and low-density lipoprotein cholesterol (LDL) levels in mice (69). This may be related to the insulinotropic properties of BCAA (especially Leu), short-term (4 weeks) BCAA-restricted diet decreased postprandial insulin secretion, increased postprandial insulin sensitivity and mitochondrial metabolism efficiency in adipose tissue (70). On the other hand, Long-term (60 weeks) supplementation with amino acids has also been shown to improve glycemic control and insulin sensitivity in older non-obese (BMI within 19∼23) T2DM patients (71). Since protein degradation is commonly increased in populations with poor IR and T2DM control (72), and the provision of additional BCAAs in the diet can mitigate protein degradation (73), it is necessary to tailor nutritional programs to different populations. In addition, valine and isoleucine are major contributors to the production of cyclic odd-chain FA (74), among which valine not only promotes α oxidation by activating PPARα, but also promotes odd-chain FA production by providing PrCoA as a substrate (75).

Different organs also show differences in amino acid profiles, liver but not muscle IR was associated with increased levels of leucine and tyrosine, leucine deprivation enhances insulin sensitivity by increasing AMPK phosphorylation and inhibiting the mTOR/S6K pathway in the mouse liver (76), while both showed higher levels of isoleucine and alanine and lower levels of glycine (48). In the liver, BCAA supplementation activates mTORC1 and suppresses mTORC2, blocks insulin-mediated Akt2 phosphorylation, and promotes its ubiquitination and degradation, negative regulation of Akt2 increases FoxO1-mediated gluconeogenesis and inhibits liver lipogenesis mediated by the sterol-regulatory element binding protein (SREBP)1/INSIG2a signaling pathway (77). It is believed that when FA are excessive, the accumulation of metabolic intermediates of BCAA (rather than BCAA itself) can competitively “block” FA β oxidation flux, resulting in the accumulation of BCAA and incomplete oxidation products of FA, leading to a corresponding decrease in glucose utilization (37). All of these suggest that the increase of BCAA in T2DM is likely to be a downstream effect caused by obesity and IR, and then plays a further mediating role in disease development (37).

Studies have suggested that increased IR leads to increased levels of circulating fasting BCAA and inflammation (78, 79). Obesity and T2DM reduce the activity of metabolic enzymes involved in BCAA catabolism, leading to BCAA accumulation (80). BCAA catabolism involves the first transamination of BCAA aminotransferase (BCAT) to branch alpha-ketoic acid (BCKA), followed by decarboxylation of BCKA by BCKA dehydrogenase complex (BCKDC), which is activated by dephosphorylation of PPM1K phosphatase and deactivated by phosphorylation of BCKD kinase. The expression of BCAT and BCKDC is relatively low in the liver, where adipose tissue and skeletal muscle are major sites of BCAA oxidative metabolism (81, 82). In human and animal models of metabolic syndrome, hypoxia, inflammation, and ER stress in adipose tissue can lead to a significant decrease in the level of BCAA catabolic enzyme (83, 84), and the accumulation of BCAA directly inhibits the activity of pyruvate dehydrogenase (PDH) and reduces the oxidative metabolism of glucose and FA (85). Another study suggested that reduced oxygenation in adipose tissue inhibited BCAA catabolism (86). Oxygen partial pressure in subcutaneous adipose tissue was negatively correlated with the expression of markers of inflammation and fibrosis. Meanwhile, hypoxia inhibited the catabolism and oxidation of BCAA, resulting in increased plasma BCAA concentration, thus promoting IR. Surgical weight loss interventions can reverse the increase in plasma concentrations by improving BCAA metabolism in adipose tissue, suggesting that changes in plasma BCAA reflect IR or relative insulin deficiency in obesity (87). Muscle biopsies in patients with T2DM also showed decreased expression of two enzymes necessary for valine and isoleucine metabolism (88). In contrast, increased BCAA catabolism effectively reduced plasma BCAA levels in T2DM patients, significantly improved peripheral glucose utilization, and increased pyruvate mitochondrial oxidation flux by 10% in muscle (89).

In addition to BCAA and AAA, multiple additional amino acids and derivatives are associated with diabetes progression (49, 90, 91). Studies have shown that glutamate is significantly increased in people with T2DM, while glutamine and glycine are associated with a 15% and 11% reduction in diabetes risk, respectively (52). After adjusting for BMI, concentrations of aspartic acid, asparagine, and histidine were strongly correlated with the incidence of prediabetes. For every 1 standard deviation increase in baseline aspartic acid and asparagine levels, the risk of prediabetes increased by 2.72 times, while for every 1 standard deviation increase in baseline histidin level, the risk of prediabetes decreased by 10% (47). This may be related to histidine’s role in regulating gluconeogenesis and anti-inflammatory (92).

Studies have shown that alanine, tryptophan, and trytophan-related metabolic intermediates are associated with a higher risk of T2DM and prediabetes (40, 43, 44, 93), and that kynurenine is the main metabolic intermediate of tryptophan, chronic inflammatory can induce activation of the tryptophan/kynurenine metabolic pathway (94), and may mediate the increased mortality associated with inflammation in T2DM (95). Circulating kynurenine levels are also affected by dietary tryptophan intake. As a result, it has been suggested that the ratio of kynurenine/tryptophan can reflect the metabolic status of tryptophan further than that of tryptophan or kynurenine concentration alone (96). This partly explains why the level of tryptophan increases at the beginning of T2DM and reverses as the disease progresses (45), and why there is no significant association between the plasma kynurenine/tryptophan ratio and T2DM risk, but the urine kynurenine/tryptophan rate is strongly associated with T2DM risk (96). In addition, indolepropionate, a tryptophan-breaking metabolite derived from the gut microbiome, was negatively associated with T2DM risk, while increased indolelactate was associated with higher T2DM risk (44, 53).

Elevated levels of lysine and its metabolic intermediate 2-aminoadipic acid were associated with an increased risk of T2DM. 2-aminoadipic acid metabolism occurs primarily in mitochondria and is broken down into acetyl-CoA before entering the (tricarboxylic acid, TCA) cycle. Plasma 2-Aminoadipic acid level increases by 47% in obesity and is positively correlated with IR (97). As a novel biomarker to predict the risk of T2DM, 2-aminoadipic acid, independent of common BCAA and AAA, has been shown to increase in concentration 12 years before the onset of diabetes symptoms (98), and as a biomarker to predict childhood obesity and related metabolic disorders 2 years later (99). In addition, the level of circulating 2-Aminoadipic acid is significantly negatively correlated with HDL, which is closely associated with cardiovascular complications such as atherosclerosis and coronary artery calcification (100, 101).

### 2.3 Lipids and acylcarnitines

Elevated blood levels of triacylglycerols (TAGs) are traditional risk indicators for T2DM (48, 102, 103). Free fatty acids (FFA) are non-esterified fatty acids in the serum that comes primarily from the breakdown of TAG. When caloric intake exceeds the normal storage and consumption capacity of lipids, fatty acids “spillover” will result in increased FFA (104). Elevated fasting FFA is associated with a three-fold increased risk of impaired glucose tolerance or T2DM over the next 5∼8 years (105). After clinical intervention, the level of FFA can also serve as an effective prognostic evaluation index (106). FFA can be divided into saturated fatty acids (SFA), monounsaturated fatty acids (MUFA), and polyunsaturated fatty acids (PUFA) according to the difference of hydrocarbon saturation. Serum FFA variations of different types are generally suggestive of hyperglycemia or T2DM (**Table 2**). Specifically, increased levels of partial n-6, n-7 and n-9 were significantly positively correlated with elevated blood glucose, while some n-3 PUFA was significantly inversely correlated with T2DM (107, 108, 113, 116). In addition, different chain lengths of SFA have particular metabolic and biological effects. Increased circulating concentrations of C15:0, C17:0 and C24:0 and very long chain of SFA are associated with lower risk of T2DM (114, 117), while C14:0, C16:0, C16:1, and C18:0 are positively correlated with T2DM risk (109, 111).

**Table 2:** Association of lipids profiles with T2DM in cohort studies.

Sample size (incident cases)	Duration of follow-up(years)	Platform	Metabolites	End point	OR/HR/RR (95% CI)	Ref
91	2	LC-MS	LPC18:2↓	T2DM	0.69^a^	(41)
151	9.5	UHPLC-MS/MS	linoleoyl-glycerophosphocholine↓	T2DM	0.67^a^	(50)
540	—	NMR	TAG↑	HOMA-IR	—	(48)
152	1-3	LC-MS	Carnitine (C3, C4, C5)↑ LPC (18:1, 22:6), SM16:0, Carnitines (C9, C10:2, C18, C18:1OH, C18:2), LPE16:0, lysophosphatidylethanolamine C16:0, acetylcholine↓	HOMA-IR	—	(49)
189	12	LC-MS MS-MS	TAG (44:1, 46:1, 48:0, 48:1, 50:0, 52:1), PC (34:2, 26:2), LPE18:2↑ TAG (56:9, 58:10, 60:12), PC38:6, LPC22:6↓	T2DM	1.35-1.94^a^ 0.67-0.78^a^	(102)
189/364	4.4/3.8	HPLC-MRM	LPl16:1, PC34:3, TAG50:2(16:2), TAG51:0(17:0), TAG54:7(22:6) ↑ PE38:4p(18:0p/20:4) ↓	T2DM	—	(103)
12132	16	GC	n-3 (EPA20:5), n-6 (GLA18:3, DGLA20:3, AA20:4, DTA22:4, DPA22:5)↑ n-3 (ALA18:3, DPA22:5, DHA22:6), n-6 (LA18:2)↓	T2DM	1.02-1.46^b^ 0.80-0.95^b^	(107)
276	4.5	NMR	Glycerol, FFAs, total TAG, MUFAs, SFA (n-7, n-9)↑ n-6 FAs↓	T2DM	1.09-1.26^a^ 0.92^a^	(108)
12132	16	GC	SFA (14:0, 16:0, 18:0)↑ SFA (15:0, 17:0, 20:0, 22:0, 23:0, 24:0)↓	T2DM	1.06-1.26^b^ 0.58-0.92^b^	(109)
507	6	LC-MS	Carnitine (C0, C3DC, C8:1, C10, C14OH, C14:1OH), acylcarnitines (C16:1, C16:2, C18, C18OH, C18:1, C18:2, C20, C20:4)↑ 3-dehydroxycarnitine, 3-dehydrocarnitine, dicarboxylic (C10DC, C12DC), acylcarnitines (C12, C12OH, C12:1) ↓	T2DM	2.48-9.41^c^ (models)	(110)
703	11	GC	C16:0, C16:1↑	T2DM	1.15-1.24^b^	(111)
250	3.8	LC-MS	TAG, DAG, PE↑ LP, PC-PL, SM, CE↓	T2DM	1.45-1.58^b^ 0.67-0.78^b^	(112)
71	5.9	GC-MS	C20:3n-6↑	T2DM	1.53^b^	(113)
284	10	GC-MS	SFA (C20:0, 22:0, 24:0)↓	Diabetes	0.68-0.99^b^	(114)
251	3.8	LC-MS	Carnitine C4OH	T2DM	1.44^b^	(115)

T2DM, Type 2 diabetes mellitus; GC, gas chromatography; GLA, γ-linolenic acid; DGLA, dihomo-γ-linolenic acid; DTA, docosatetraenoic acid; n-6 DPA, docosapentaenoic acid; ALA, α-linolenic acid; SFA, saturated fatty acid; LC-MRM, liquid chromatography multiple reaction monitoring; HPLC, high-performance liquid chromatography; MS-MS, tandem mass spectrometry; CE, cholesteryl ester; PC, phosphatidylcholine; LPl, lyso-phosphatidylinositol; PPPE, polyunsaturated plasmalogen phosphatidylethanolamine; LPE, lysophosphatidylethanolamines; LPC, lysophosphatidylcholines; SM, sphingomyelin; TAG, triacylglycerol; DAG, diacylglycerol; PE, phosphatidylethanolamine; LP, lysophospholipid; PC-PL, phosphatidylcholine-plasmalogen; CE, cholesterol ester; ↑, increased; ↓, decreased; —, not available, ^a^, odd ratio (OR); ^b^, hazard ratio (HR); ^c^, relative risk (RR).

Increased FFA release and oxidation rate can antagonize glucose oxidation, resulting in the disturbance of pyruvate metabolism and impaired insulin sensitivity (118, 119), and this damage to glucose homeostasis by FFA is commonly referred to as lipotoxicity. Extensive evidence has demonstrated the role of lipotoxicity in IR and pancreatic β-cell injury (120, 121). Elevated plasma FFA levels are also known to cause TAG and DAG deposition in a variety of tissues and organs, gradually accumulating DAG enhances NADPH oxidase activity through the PKC pathway, exacerbates oxidative stress and cytokine transcription, and promotes cell differentiation, proliferation, and apoptosis. Activation of the PKC pathway is a critical mechanism leading to diabetes cardiovascular disease (122). Thus, lipid management is absolutely recommended for the prevention and treatment of vascular complications of T2DM.

**Figure 3:**
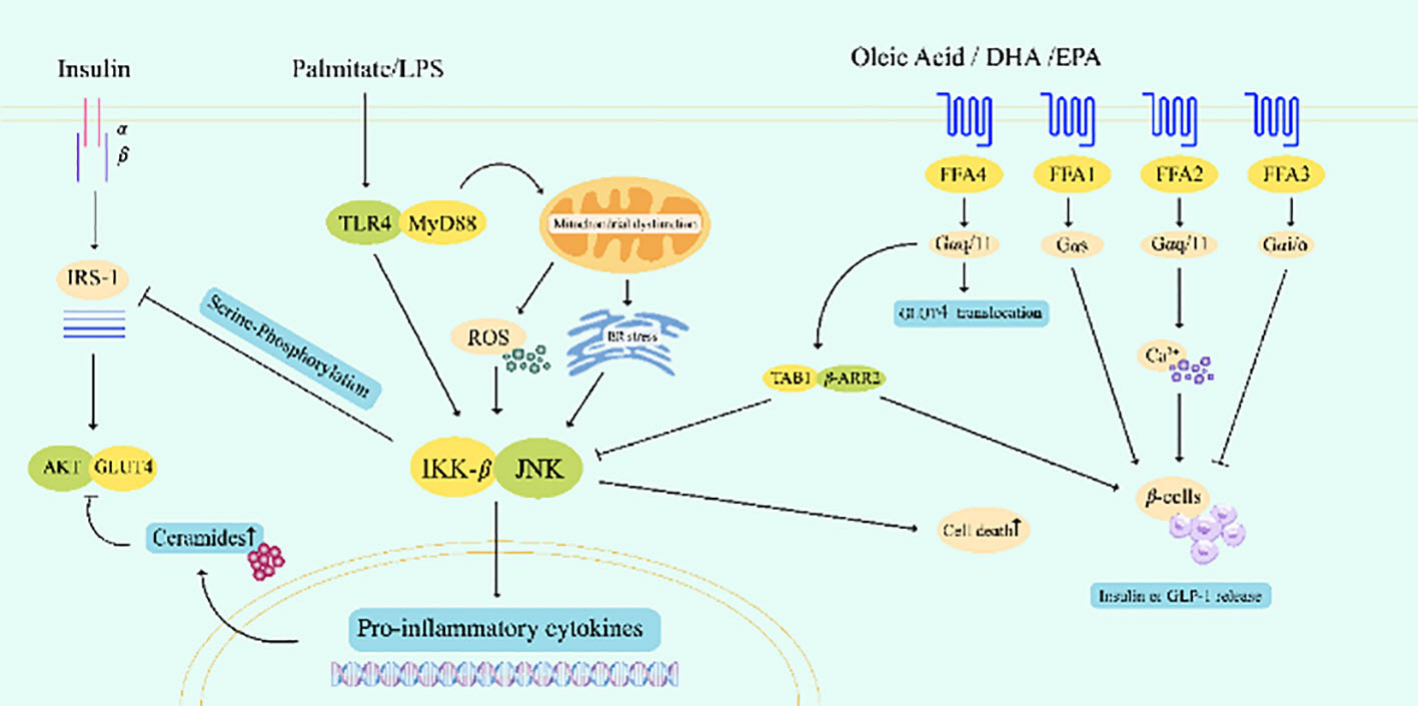
Palmitate cooperated with LPS to amplify TLR4-related signaling pathways, directly or indirectly inhibited insulin signaling, and caused β-cell apoptosis in islets. The activation of FFA4 by certain PUFAs such as oleic acid, DHA and EPA can competitively bind TAB1 through the recruitment of β-arrestin-2, inhibit the phosphorylation and activation of TAK1, inhibit the pro-inflammatory response, and promote the release of insulin and GLP-1. LPS, lipopolysaccharide; IRS-1, insulin receptor substrate 1; TLR4, toll-like receptor 4; AKT, protein kinase B; GLUT, glucose transporter; ROS, reactive oxygen species; IKK-β, inhibitor kappa B kinase-β; JNK, Jun N-terminal kinase; DHA, docosahexaenoic acid; EPA, eicosapentaenoic acid; FFA, free fatty acid receptor; TAB1, TAK1 binding protein 1; β-ARR2, β-arrestin-2; GLP-1, glucagon-like peptide-1.

The inflammatory response mediated by SFA is an essential cause of IR and β-cell damage (**Figure 3**). SFA and lipopolysaccharide synergistically amplifies the effects of decreased β-cell viability, increased apoptosis, and decreased basal insulin secretion, and significantly alleviates lipid-induced β-cell damage by blocking toll-like receptor 4 (TLR4) or overexpressing neutral ceramidase (NCDase) activity (123, 124). Palmitate (C16:00) is the most abundant SFA in dietary and plasma, palmitate can enhance the interaction between TLR and myeloid differentiation primary response protein MyD88, mediating β-cell death (125). On the one hand, it directly inhibits insulin signal transduction by activating the phosphorylation of Jun N-terminal kinase (JNK) and the inhibitor of nuclear factor-κB (NF-κB) kinas (IKKβ). On the other hand, degradation of inhibitor of NF-κB (IκB) leads to nuclear translocation of NF-κB, this increases cytokine transcription and exacerbates inflammation. TLR4, IKKβ, or JNK knockout inhibited the expression of inflammatory cytokines in adipocytes and macrophages and protected mice against lipid-induced IR (126–128). In contrast, some PUFA such as docosahexaenoic acid (DHA) inhibit the production of TLR4-induced inflammatory cytokines (129), improve insulin sensitivity and insulin secretion capacity to some extent, and reduce the risk of T2DM (128, 130).

In specific lipids and derivatives, baseline lysophosphatidylcholine (LPC), phosphatidylcholine (PC), sphingomyelin, and cholesterol esters were inversely associated with T2DM risk (112). LPC were strongly associated with IR and β-cell dysfunction (50). Decreased concentrations of LPC18:2 and 16:0 are associated with the onset of T2DM (41, 131), and increased levels of PC O-16:1/0:0, (O-18:1/0:0)/(P-18:0/0:0) and LPC 20:2 can increase the 10-year risk of T2DM by 29% (132). Diacylphosphatidylcholine C32:1, C36:1, C38:3 and C40:5 were positively associated with T2DM risk. PC O-20:0/O-20:0, 22:6/20:4, LPC18:0, sphingomyelin C16:1 and acyl-alkyl-phosphatidylcholine C34:3, C40:6, C42:5, C44:4 and C44:5 were negatively correlated with T2DM risk (133–135). In the early stages of dysglycemia and IR, fasting concentration of linoleoylglycerophosphocholine is decreased, independent of classical predictors, as an indicator of worsening glucose tolerance (50).

Ceramide is a relatively minor component of the total cellular lipidome with a particularly low abundance, and increased ceramide content has been shown to be positively correlated with HOMA-IR, fasting glucose, and cardiovascular diseases (136, 137). Elevated ceramides are key lipotoxic species in skeletal muscle, liver, adipose tissue, and vascular cells, and contribute to disease progression by interfering with insulin signaling, stimulating lipid uptake, and enhancing inflammatory cytokines (138–142). Of the different types of sphingolipids, C16 ceramides and C18 ceramides are more damaging to adipose and liver function. C16:0 ceramides can impair mitochondrial oxidative phosphorylation by inhibiting mitochondrial complex II and promoting mitochondrial fission, reduce mitochondrial respiration, and promote the release of cytochrome c to induce apoptosis by increasing the permeability of mitochondrial outer membrane (139, 143). Inhibition of ceramide synthesis can improve insulin sensitivity and prevent obesity-induced diabetes (144). It also increases brown adipocyte numbers, mitochondrial activity, and promotes the polarization of adipose tissue macrophages towards the M2 anti-inflammatory phenotype (145).

Acylcarnitines, metabolites of FA, play crucial roles in cellular energy metabolism and are gradually considered as influential biomarkers of metabolic disorders in metabolic syndrome, diabetes, cardiovascular diseases and other diseases. For example, C2, C3DC-CH3, C4, C5, C7 and C26 have been observed to be associated with HOMA-IR (49). In cross-sectional studies, acylcarnitines were elevated in IGT and diabetic individuals (146), reflecting incomplete fatty acid beta oxidation in the organism, but acetylcarnitine C2 did not predict IGT or T2DM years before onset, so it is more likely to be a quick-acting event (41).

Recently, FFA ligand-specific G-protein-coupled receptor (GPR) including GPR40 (also known as FFA1), GPR43 (FFA2), GPR41 (FFA3), GPR120 (FFA4) has been extensively studied. In HFD-fed mice, FFA2 function is more mediated by Gi/o and activated by short-chain FA, which inhibit insulin signaling in adipose tissue, increasing energy expenditure and improving insulin sensitivity in different tissues, including liver and muscle (147, 148). Propionic acid (C3) and valeric acid (C5) can increase basal glucose uptake in adipocytes and muscle cells by activating FFA3, while this effect is decreased after FFA3 inhibition (149).

FFA1 and FFA4, as long chain FFA receptors, are expressed in a variety of tissues and cells, such as adipocytes, macrophages, and pancreatic β-cells. FFA1 activation alone or synergistically amplifies glucose-dependent insulin secretion by affecting cellular Ca^2+^ signaling and increasing intracellular Ca^2+^ concentration (150, 151), which is critical for maintaining the homeostasis of glucose and lipid metabolism in IR individuals, making it an attractive research target for the regulation of glucose and lipid metabolism (152, 153). However, the exact mechanism of FFA1 in β-cells is still under debate. Although FFA1 mediates insulin secretion in response to acute FFA exposure, long-term activation of FFA1 is also involved in lipotoxicity to β-cells. The diversity of FFAs may partly explain the difference in efficacy, for example, palmitate increases endoplasmic reticulum stress and mitochondrial dysfunction through FFA1 activation (154–157), resulting in increased apoptosis and decreased insulin secretion. In addition, FFAR1 also responds to trans isomers of conjugated linoleic acid or arachidonic acid (158), regulating the crosstalk between Akt/mTOR and IRS-1 signaling in β-cells under lipotoxicity conditions, promoting the progression of IR and T2DM (159). Oleic acid can inhibit the activation of JNK and NF-κB, inhibit inflammatory cytokine secretion, and improve insulin sensitivity (160), while palmitoleic acid can reverse the HFD-induced proinflammatory polarization of macrophages by activating AMPK and FFA4 (161, 162), independently of the PPAR-α mechanism (163). n-3 PUFA activates FFA4, which inhibits inflammation and increases insulin sensitivity (162). This is at least in part through the regulation of NOD-like receptor family pyrin domain-containing 3 (NLRP3) inflammasome and macrophage conversion to the M2 anti-inflammatory standard (164), and FFA4 also mediates a variety of effects such as glucagon-like peptide-1 (GLP-1) secretion, islet function, and appetite control (165, 166).

### 2.4 Others

α-hydroxybutyrate (α-HB) is an organic acid derived from α-ketobutyrate, a by-product of amino acids such as methionine and threonine catabolism and glutathione synthesis (167). Increasing evidence has shown that α-HB is an early predictor of IR and impaired glucose tolerance (168, 169), the combination of α-HB and L-glycerophosphate choline showed similar accuracy to glucose in OGTT assay, and the plasma level of α-HB was negatively correlated with insulin sensitivity (**Table 3**). It has also been shown to be associated with β-cell dysfunction in statistical and mechanism studies (50, 51, 170). Since glutathione is a vital antioxidant, which can inhibit oxidative damage caused by an imbalance of lipid peroxides and free radicals in cells, the potential mechanism of increased α-HB may be a compensatory increase in liver glutathione synthesis flux after body REDOX dysplasia (169).

**Table 3:** Association of metabolic intermediate profiles with T2DM in cohort studies.

Sample size (incident cases)	Duration of follow-up (years)	Platform	Metabolites	End point	OR/HR (95% CI)	Ref
9180	5.7	LC-MS	Kynurenine, kynurenate, xanthurenate, quinolinate, indolelactate**↑** indolepropionate↓	T2DM	—	(44)
251	3.8	LC-MS	Quinolinic acid**↑**	T2DM	1.39^b^	(45)
350	12	LC-MS/MS	2-aminoadipic acid**↑**	T2DM	1.59^a^	(98)
16	2	NMR	α-Hydroxybutyrate**↑**	IR	—	(51)
151	9.5	HPLC-MS/MS	α-Hydroxybutyrate**↑**	T2DM	1.26^a^	(50)
152	1-3	LC-MS	*N*-acetyl-tryptophan, methyladenosine, N-acetyl-leucine, dimethylglycine, hypoxanthine, thiamin**↑** Betaine, guanidoacetic acid, β-Amino-isobutyric acid↓	HOMA-IR	—	(49)

T2DM, type 2 diabetes mellitus; IR, insulin resistance; LC-MS, liquid chromatography-mass spectrometry; HOMA-IR, homeostasis model assessment for insulin resistance; HPLC, high-performance liquid chromatography; MS/MS, tandem mass spectrometry; NMR, nuclear magnetic resonance; ↑, increased; ↓, decreased; —, not available, ^a^, odd ratio (OR); ^b^, hazard ratio (HR).

3-Hydroxyisobutyrate (3-HIB), an intermediate product of valine decomposition, is considered a higher sensitive biomarker of T2DM than valine. Impaired valine catabolism increases 3-HIB production, leading to increased lipid oxidation and acylcarnitine accumulation. Currently, 3-HIB is believed to mediate the occurrence of IR by affecting FA uptake in endothelial cells, and 3-HIB treatment has a regulatory effect on mitochondrial metabolism in white and brown adipocytes (171). Recent studies have shown that changes in the gut microbiome are involved in the metabolic disorders of T2DM, where imidazole propionate (ImP) is a product of histidine microbial metabolism. Serum ImP expression is upregulated in T2DM patients due to changes in microbial metabolism rather than histidine intake per se (172). It affects insulin receptor substrates and inflammatory signals by activating p38γ/MAPK/p62/mTORC1 signals, leading to impaired glucose metabolism (173). In addition, ImP (but not the precursor histidine) also showed a significant association with diastolic blood pressure in the overweight/obesity population, showing a possible role in CVD complications (174).

Serum concentrations of 3-carboxy-4-methyl-5-propyl-2-furan propionic acid (CMPF), the main endogenous metabolite of furan FA, are elevated in patients with impaired glucose tolerance and T2DM, and can directly act on pancreatic β-cells to lead to impaired insulin secretion (175–177). However, one study showed the opposite result: compared with the control group, the concentration of serum CMPF in the T2DM group was lower and was negatively correlated with the changes in serum TAG (178), although this study was limited by the small sample size, it still suggested that the metabolism of CMPF might be affected by race, diet and other factors. Supplementation of n-3 FA can increase the level of CMPF, and there is a positive correlation between docosahexaenoic acid (DPA) and DHA levels and CMPF, but no significant relationship between eicosapentaenoic acid (EPA) (179). It should be noted that although exogenous n-3 FA intake (e.g. fish) can significantly increase circulating CMPF concentration, it is still far below the level of T2DM and low doses of CMPF do not have a significant effect on glucose metabolism (180).

Bile acids (BAs) can act as signaling regulators for lipids and glucose metabolism, and the concentration of BAs changed has been linked to metabolic disorders such as IR. Studies have shown that after adjusting for age, sex, BMI, waist circumference, and fasting blood glucose, increased circulating 12α-hydroxylated BAs concentration is significantly associated with increased HOMA-IR and fasting blood glucose (132, 181), but it cannot be used as an effective predictor of diabetes (182, 183). This may be because decreased insulin sensitivity and impaired glucose tolerance occur before the rise in BAs. The increase in circulating BAs is not the factor that causes the change in glucose metabolism, but its downstream effect (184). Recent study has also confirmed that the increase of circulating BAs in T2DM individuals is positively correlated with fasting blood glucose, HbA1c, and HOMA-IR, which may be due to insulin signaling dysfunction. However, insulin treatment did not significantly affect the total level of BAs. Therefore, more studies are needed on the composition of BAs and its role as a regulator in metabolic disorders (185).

## 3 Effects of anti-hyperglycemic medications on metabolites

Many of commonly used anti-hyperglycemic medications can also have pleiotropic effects on the metabolite profile, which may positively affect T2DM and complications. In the following, we summarize the therapeutic effects of current mainstream hypoglycemic drugs on metabolites associated with different diabetes risks.

### 3.1 Metformin

Metformin is a common drug for T2DM management. In randomized controlled trials, taking metformin was associated with increased levels of betaine, alanine, histidine, leucine/isoleucine and decreased levels of carnitine, phenylalanine, tyrosine and valine (186, 187). In terms of blood lipids, metformin was associated with an increase in TAG of higher carbon numbers (188), and elevated levels of the latter seemed to predict a reduced risk of T2DM (102), and very-low-density lipoprotein (VLDL)-triglyceride levels were significantly reduced (189). In another small sample size study, T2DM patients treated with metformin had increased serum trimethylamine-N-oxide, α-HB, and tryptophan, while acetoacetic acid, phenylalanine, and LPC (16:0, 18:0, and 18:2) were decreased (190).

### 3.2 Thiazolidinedione

Compared with metformin, pioglitazone increased myocardial glucose uptake and decreased hepatic TAG content (191, 192), but did not show any effect on subcutaneous fat volume. Compared with rosiglitazone, pioglitazone has a smaller increase in LDL and a larger increase in HDL, and promotes the transformation of VLDL to larger LDL by reducing asymmetric dimethylarginine levels (193, 194), which has a protective effect on cardiovascular diseases.

### 3.3 GLP−1 receptor agonist

Treatment with liraglutide can significantly reduce serum tyrosine, valine and isoleucine levels in obese people, but has no significant effect on T2DM patients (195, 196). After liraglutide treatment, ceramides, phospholipids, hexocyl-ceramides, LPC, sphingolipids, and TAG were significantly deregulated in T2DM patients, demonstrating the cardiovascular system benefits of liraglutide (197–200). Exenatide treatment for 6 months was effective in reducing body weight, cysteine, and FFA concentration, while levels of aminoisobutyric acid, anandamide, and sarcosine tended to increase (201, 202). The efficacy of duraglutide was also associated with a significant reduction in 2-hydroxybutyric acid and a significant upregulation of threonine compared to placebo (203). ​In addition, high doses of trusted downregulated BCAA, glutamate, 3-hydroxyisobutyrate, branched-chain ketoacids, and 2-hydroxybutyrate (204).

### 3.4 DPP-4 inhibitor

Studies have shown that 6 months of vildagliptin treatment can reduce the level of asymmetric dimethylarginine in T2DM patients (205), but has no significant effects on FFA, glycerol, lactic acid and pyruvate (206, 207).

### 3.5 Concomitant drugs

The present investigation shows that 3 months of metformin plus pioglitazone can significantly reduce the levels of phenylalanine/tyrosine, citrulline/arginine, and lysine/α-aminoadipic acid in T2DM and obese adults (208). Compared with treatment alone, the combination of pioglitazone and exenatide reduced hepatic fat and plasma TAG more significantly (209).

At present, the effects of hypoglycemic drugs on serum metabolites in patients with T2DM are more focused on the effects of lipids and lipoproteins, but the number of studies on amino acids and metabolic derivatives are limited. GLP-1 agonists have shown relatively better effects on lipid and amino acid metabolites, and improvements in metabolites associated with cardiovascular risk have been observed in short-term trials, but long-term follow-up evidence is still lacking. An anti-hyperglycemic drug’s effect on blood metabolites needs more prospective, intervention and randomized clinical trial studies to confirm the molecular mechanism of further metabolites.

## 4 Perspective

As a typical metabolic disease, exploring changes in metabolites and their regulatory mechanisms is closer to the essence of T2DM. ​Among the promising metabolites, blood concentrations of hexose, BCAA, AAA, TAG, phospholipids and sphingomyelins were significantly and positively associated with T2DM incidence, while glycine and glutamine were negatively associated with T2DM risk. However, using only one metabolite type as a biomarker has many limitations in terms of disease duration, race, or diet, so a comprehensive judgment of multiple metabolite prediction models is necessary. Understanding the metabolism of metabolites in specific tissues and the influence of the regulation of corresponding receptors on immune response and biological efficacy, as well as verifying causality through mechanism studies, is key to metabolite research. Finally, while we have an initial understanding of the functions of metabolites as regulators, the results of dietary interventions do not completely match our expectations. How dietary nutrients cause changes in metabolic pathways and certain protein signaling pathways, as well as the role of gut flora in metabolite synthesis and downstream regulation, will be attractive topics.

## Author contributions

YL mainly wrote the manuscript. DW ccollects data, corrects and provides fund support. Y-PL reviews, editing and provides fund support. All authors have read and agreed to the published version of the manuscript.

## References

[1] Magliano DJ Boyko EJ committee IDFDAtes. IDF Diabetes Atlas. Idf diabetes atlas. Brussels: International Diabetes Federation (2021).

[2] White PJ McGarrah RW Grimsrud PA Tso SC Yang WH Haldeman JM. The BCKDH kinase and phosphatase integrate BCAA and lipid metabolism via regulation of ATP-citrate lyase. Cell Metab (2018) 27(6):1281–93.e7. doi: 10.1016/j.cmet.2018.04.015 29779826 PMC5990471

[3] Katz LS Brill G Zhang P Kumar A Baumel-Alterzon S Honig LB. Maladaptive positive feedback production of ChREBPβ underlies glucotoxic β-cell failure. Nat Commun (2022) 13(1):4423. doi: 10.1038/s41467-022-32162-x 35908073 PMC9339008

[4] Segerstolpe Å Palasantza A Eliasson P Andersson EM Andréasson AC Sun X. Single-cell transcriptome profiling of human pancreatic islets in health and type 2 diabetes. Cell Metab (2016) 24(4):593–607. doi: 10.1016/j.cmet.2016.08.020 27667667 PMC5069352

[5] Hou J Li Z Zhong W Hao Q Lei L Wang L. Temporal transcriptomic and proteomic landscapes of deteriorating pancreatic islets in type 2 diabetic rats. Diabetes (2017) 66(8):2188–200. doi: 10.2337/db16-1305 28559245

[6] Haythorne E Rohm M van de Bunt M Brereton MF Tarasov AI Blacker TS. Diabetes causes marked inhibition of mitochondrial metabolism in pancreatic β-cells. Nat Commun (2019) 10(1):2474. doi: 10.1038/s41467-019-10189-x 31171772 PMC6554411

[7] Haythorne E Lloyd M Walsby-Tickle J Tarasov AI Sandbrink J Portillo I. Altered glycolysis triggers impaired mitochondrial metabolism and mTORC1 activation in diabetic β-cells. Nat Commun (2022) 13(1):6754. doi: 10.1038/s41467-022-34095-x 36376280 PMC9663558

[8] Guasch-Ferré M Santos JL Martínez-González MA Clish CB Razquin C Wang D. Glycolysis/gluconeogenesis- and tricarboxylic acid cycle-related metabolites, Mediterranean diet, and type 2 diabetes. Am J Clin Nutr (2020) 111(4):835–44. doi: 10.1093/ajcn/nqaa016 PMC7138680 32060497

[9] Ishitobi M Hosaka T Morita N Kondo K Murashima T Kitahara A. Serum lactate levels are associated with serum alanine aminotransferase and total bilirubin levels in patients with type 2 diabetes mellitus: A cross-sectional study. Diabetes Res Clin practice (2019) 149:1–8. doi: 10.1016/j.diabres.2019.01.028 30711436

[10] Santos JL Cataldo LR Cortés-Rivera C Bravo C Díaz-Casanova L Martínez JA. Plasma lactate and leukocyte mitochondrial DNA copy number as biomarkers of insulin sensitivity in non-diabetic women. J Physiol Biochem (2019) 75(3):285–97. doi: 10.1007/s13105-019-00672-w 30868510

[11] Memon B Elsayed AK Bettahi I Suleiman N Younis I Wehedy E. iPSCs derived from insulin resistant offspring of type 2 diabetic patients show increased oxidative stress and lactate secretion. Stem Cell Res Ther (2022) 13(1):428. doi: 10.1186/s13287-022-03123-4 35987697 PMC9392338

[12] Russo S Kwiatkowski M Govorukhina N Bischoff R Melgert BN. Meta-inflammation and metabolic reprogramming of macrophages in diabetes and obesity: the importance of metabolites. Front Immunol (2021) 12:746151. doi: 10.3389/fimmu.2021.746151 34804028 PMC8602812

[13] Ranganathan P Shanmugam A Swafford D Suryawanshi A Bhattacharjee P Hussein MS. GPR81, a cell-surface receptor for lactate, regulates intestinal homeostasis and protects mice from experimental colitis. J Immunol (Baltimore Md: 1950) (2018) 200(5):1781–9. doi: 10.4049/jimmunol.1700604 PMC5858928 29386257

[14] Li X Yang Y Zhang B Lin X Fu X An Y. Lactate metabolism in human health and disease. Signal transduction targeted Ther (2022) 7(1):305. doi: 10.1038/s41392-022-01206-5 PMC9434547 36050306

[15] Hirschhaeuser F Sattler UG Mueller-Klieser W. Lactate: a metabolic key player in cancer. Cancer Res (2011) 71(22):6921–5. doi: 10.1158/0008-5472.CAN-11-1457 22084445

[16] McCarthy SF Islam H Hazell TJ. The emerging role of lactate as a mediator of exercise-induced appetite suppression. Am J Physiol Endocrinol Metab (2020) 319(4):E814–e9. doi: 10.1152/ajpendo.00256.2020 32893673

[17] Carrière A Lagarde D Jeanson Y Portais JC Galinier A Ader I. The emerging roles of lactate as a redox substrate and signaling molecule in adipose tissues. J Physiol Biochem (2020) 76(2):241–50. doi: 10.1007/s13105-019-00723-2 31898016

[18] Chen AN Luo Y Yang YH Fu JT Geng XM Shi JP. Lactylation, a novel metabolic reprogramming code: current status and prospects. Front Immunol (2021) 12:688910. doi: 10.3389/fimmu.2021.688910 34177945 PMC8222712

[19] Yang K Holt M Fan M Lam V Yang Y Ha T. Cardiovascular dysfunction in COVID-19: association between endothelial cell injury and lactate. Front Immunol (2022) 13:868679. doi: 10.3389/fimmu.2022.868679 35401579 PMC8984030

[20] Bae KH Choi YK Bae KH Bae KH Bae KH. Role of lactate dehydrogenase a (LDH-A) in diabetic vascular complication. J Diabetes. doi: 10.1530/endoabs.49.EP383

[21] Zhu Y Han XQ Sun XJ Yang R Ma WQ Liu NF. Lactate accelerates vascular calcification through NR4A1-regulated mitochondrial fission and BNIP3-related mitophagy. Apoptosis. doi: 10.1007/s10495-020-01592-7 31993850

[22] Young A Oldford C Mailloux RJ. Lactate dehydrogenase supports lactate oxidation in mitochondria isolated from different mouse tissues. Redox Biol (2020) 28:101339. doi: 10.1016/j.redox.2019.101339 31610469 PMC6812140

[23] San-Millan I Sparagna GC Chapman HL Warkins VL Chatfield KC Shuff SR. Chronic lactate exposure decreases mitochondrial function by inhibition of fatty acid uptake and cardiolipin alterations in neonatal rat cardiomyocytes. Front Nutr (2022) 9:809485. doi: 10.3389/fnut.2022.809485 35308271 PMC8931465

[24] Mizukami H Osonoi S. Pathogenesis and molecular treatment strategies of diabetic neuropathy collateral glucose-utilizing pathways in diabetic polyneuropathy. Int J Mol Sci (2020) 22(1):94. doi: 10.3390/ijms22010094 33374137 PMC7796340

[25] Allalou A Nalla A Prentice KJ Liu Y Zhang M Dai FF. A predictive metabolic signature for the transition from gestational diabetes mellitus to type 2 diabetes. Diabetes (2016) 65(9):2529–39. doi: 10.2337/db15-1720 PMC5001181 27338739

[26] Fiehn O Garvey WT Newman JW Lok KH Hoppel CL Adams SH. Plasma metabolomic profiles reflective of glucose homeostasis in non-diabetic and type 2 diabetic obese African-American women. PloS One (2010) 5(12):e15234. doi: 10.1371/journal.pone.0015234 21170321 PMC3000813

[27] Bian C Wang Y Li J Gao J Luan Z Cui X. Endogenous fructose is correlated with urinary albumin creatinine ratios and uric acid in type 2 diabetes mellitus. Diabetes Res Clin practice (2021) 179:109034. doi: 10.1016/j.diabres.2021.109034 34487756

[28] Basaranoglu M Basaranoglu G Sabuncu T Sentürk H. Fructose as a key player in the development of fatty liver disease. World J gastroenterol (2013) 19(8):1166–72. doi: 10.3748/wjg.v19.i8.1166 PMC3587472 23482247

[29] Tang WH Martin KA Hwa J. Aldose reductase, oxidative stress, and diabetic mellitus. Front Pharmacol (2012) 3:87. doi: 10.3389/fphar.2012.00087 22582044 PMC3348620

[30] Tang WH Wu S Wong TM Chung SK Chung SS. Polyol pathway mediates iron-induced oxidative injury in ischemic-reperfused rat heart. Free Radical Biol Med (2008) 45(5):602–10. doi: 10.1016/j.freeradbiomed.2008.05.003 18549825

[31] Yang Y Hayden MR Sowers S Bagree SV Sowers JR. Retinal redox stress and remodeling in cardiometabolic syndrome and diabetes. Oxid Med Cell longevity (2010) 3(6):392–403. doi: 10.4161/oxim.3.6.14786 PMC3154050 21307645

[32] Kang Q Yang C. Oxidative stress and diabetic retinopathy: Molecular mechanisms, pathogenetic role and therapeutic implications. Redox Biol (2020) 37:101799. doi: 10.1016/j.redox.2020.101799 33248932 PMC7767789

[33] Issad T Masson E Pagesy P. O-GlcNAc modification, insulin signaling and diabetic complications. Diabetes Metab (2010) 36(6 Pt 1):423–35. doi: 10.1016/j.diabet.2010.09.001 21074472

[34] Issad T Kuo M. O-GlcNAc modification of transcription factors, glucose sensing and glucotoxicity. Trends Endocrinol metabolism: TEM (2008) 19(10):380–9. doi: 10.1016/j.tem.2008.09.001 18929495

[35] Felig P Marliss E Cahill GF Jr. Plasma amino acid levels and insulin secretion in obesity. New Engl J Med (1969) 281(15):811–6. doi: 10.1056/NEJM196910092811503 5809519

[36] Würtz P Soininen P Kangas AJ Rönnemaa T Lehtimäki T Kähönen M. Branched-chain and aromatic amino acids are predictors of insulin resistance in young adults. Diabetes Care (2013) 36(3):648–55. doi: 10.2337/dc12-0895 PMC3579331 23129134

[37] Newgard CB. Interplay between lipids and branched-chain amino acids in development of insulin resistance. Cell Metab (2012) 15(5):606–14. doi: 10.1016/j.cmet.2012.01.024 PMC3695706 22560213

[38] Ramzan I Ardavani A Vanweert F Mellett A Atherton PJ Idris I. The association between circulating branched chain amino acids and the temporal risk of developing type 2 diabetes mellitus: A systematic review & Meta-analysis. Nutrients (2022) 14(20):4411. doi: 10.3390/nu14204411 36297095 PMC9610746

[39] Cosentino RG Churilla JR Josephson S Molle-Rios Z Hossain MJ Prado WL. Branched-chain amino acids and relationship with inflammation in youth with obesity: A randomized controlled intervention study. J Clin Endocrinol Metab (2021) 106(11):3129–39. doi: 10.1210/clinem/dgab538 34286837

[40] Wang TJ Larson MG Vasan RS Cheng S Rhee EP McCabe E. Metabolite profiles and the risk of developing diabetes. Nat Med (2011) 17(4):448–53. doi: 10.1038/nm.2307 PMC3126616 21423183

[41] Wang-Sattler R Yu Z Herder C Messias AC Floegel A He Y. Novel biomarkers for pre-diabetes identified by metabolomics. Mol Syst Biol (2012) 8:615. doi: 10.1038/msb.2012.43 23010998 PMC3472689

[42] Palmer ND Stevens RD Antinozzi PA Anderson A Bergman RN Wagenknecht LE. Metabolomic profile associated with insulin resistance and conversion to diabetes in the Insulin Resistance Atherosclerosis Study. J Clin Endocrinol Metab (2015) 100(3):E463–8. doi: 10.1210/jc.2014-2357 PMC4333040 25423564

[43] Tillin T Hughes AD Wang Q Würtz P Ala-Korpela M Sattar N. Diabetes risk and amino acid profiles: cross-sectional and prospective analyses of ethnicity, amino acids and diabetes in a South Asian and European cohort from the SABRE (Southall And Brent REvisited) Study. Diabetologia (2015) 58(5):968–79. doi: 10.1007/s00125-015-3517-8 PMC4392114 25693751

[44] Qi Q Li J Yu B Moon JY Chai JC Merino J. Host and gut microbial tryptophan metabolism and type 2 diabetes: an integrative analysis of host genetics, diet, gut microbiome and circulating metabolites in cohort studies. Gut (2022) 71(6):1095–105. doi: 10.1136/gutjnl-2021-324053 PMC8697256 34127525

[45] Yu E Papandreou C Ruiz-Canela M Guasch-Ferre M Clish CB Dennis C. Association of tryptophan metabolites with incident type 2 diabetes in the PREDIMED trial: A case-cohort study. Clin Chem (2018) 64(8):1211–20. doi: 10.1373/clinchem.2018.288720 PMC6218929 29884676

[46] McCormack SE Shaham O McCarthy MA Deik AA Wang TJ Gerszten RE. Circulating branched-chain amino acid concentrations are associated with obesity and future insulin resistance in children and adolescents. Pediatr Obes (2013) 8(1):52–61. doi: 10.1111/j.2047-6310.2012.00087.x 22961720 PMC3519972

[47] Owei I Umekwe N Stentz F Wan J Dagogo-Jack S. Amino acid signature predictive of incident prediabetes: A case-control study nested within the longitudinal pathobiology of prediabetes in a biracial cohort. Metabolism: Clin experimental (2019) 98:76–83. doi: 10.1016/j.metabol.2019.06.011 PMC6690793 31228482

[48] Vogelzangs N van der Kallen CJH van Greevenbroek MMJ van der Kolk BW Jocken JWE Goossens GH. Metabolic profiling of tissue-specific insulin resistance in human obesity: results from the Diogenes study and the Maastricht Study. Int J Obes (2005) (2020) 44(6):1376–86. doi: 10.1038/s41366-020-0565-z 32203114

[49] Papandreou C Bulló M Ruiz-Canela M Dennis C Deik A Wang D. Plasma metabolites predict both insulin resistance and incident type 2 diabetes: a metabolomics approach within the Prevención con Dieta Mediterránea (PREDIMED) study. Am J Clin Nutr (2019) 109(3):626–34. doi: 10.1093/ajcn/nqy262 PMC7307433 30796776

[50] Ferrannini E Natali A Camastra S Nannipieri M Mari A Adam KP. Early metabolic markers of the development of dysglycemia and type 2 diabetes and their physiological significance. Diabetes (2013) 62(5):1730–7. doi: 10.2337/db12-0707 PMC3636608 23160532

[51] Tricò D Prinsen H Giannini C de Graaf R Juchem C Li F. Elevated α-hydroxybutyrate and branched-chain amino acid levels predict deterioration of glycemic control in adolescents. J Clin Endocrinol Metab (2017) 102(7):2473–81. doi: 10.1210/jc.2017-00475 PMC5505187 28482070

[52] Guasch-Ferré M Hruby A Toledo E Clish CB Martínez-González MA Salas-Salvadó J. Metabolomics in prediabetes and diabetes: A systematic review and meta-analysis. Diabetes Care (2016) 39(5):833–46. doi: 10.2337/dc15-2251 PMC4839172 27208380

[53] Morze J Wittenbecher C Schwingshackl L Danielewicz A Rynkiewicz A Hu FB. Metabolomics and type 2 diabetes risk: an updated systematic review and meta-analysis of prospective cohort studies. Diabetes Care (2022) 45(4):1013–24. doi: 10.2337/dc21-1705 PMC9016744 35349649

[54] Xu F Tavintharan S Sum CF Woon K Lim SC Ong CN. Metabolic signature shift in type 2 diabetes mellitus revealed by mass spectrometry-based metabolomics. J Clin Endocrinol Metab (2013) 98(6):E1060–5. doi: 10.1210/jc.2012-4132 23633210

[55] Tai ES Tan ML Stevens RD Low YL Muehlbauer MJ Goh DL. Insulin resistance is associated with a metabolic profile of altered protein metabolism in Chinese and Asian-Indian men. Diabetologia (2010) 53(4):757–67. doi: 10.1007/s00125-009-1637-8 PMC3753085 20076942

[56] Zhang X Wang Y Hao F Zhou X Han X Tang H. Human serum metabonomic analysis reveals progression axes for glucose intolerance and insulin resistance statuses. J Proteome Res (2009) 8(11):5188–95. doi: 10.1021/pr900524z 19697961

[57] Menni C Fauman E Erte I Perry JR Kastenmüller G Shin SY. Biomarkers for type 2 diabetes and impaired fasting glucose using a nontargeted metabolomics approach. Diabetes (2013) 62(12):4270–6. doi: 10.2337/db13-0570 PMC3837024 23884885

[58] Würtz P Wang Q Kangas AJ Richmond RC Skarp J Tiainen M. Metabolic signatures of adiposity in young adults: Mendelian randomization analysis and effects of weight change. PloS Med (2014) 11(12):e1001765. doi: 10.1371/journal.pmed.1001765 25490400 PMC4260795

[59] Lotta LA Scott RA Sharp SJ Burgess S Luan J Tillin T. Genetic predisposition to an impaired metabolism of the branched-chain amino acids and risk of type 2 diabetes: A mendelian randomization analysis. PloS Med (2016) 13(11):e1002179. doi: 10.1371/journal.pmed.1002179 27898682 PMC5127513

[60] Saxton RA Sabatini DM. mTOR signaling in growth, metabolism, and disease. Cell (2017) 168(6):960–76. doi: 10.1016/j.cell.2017.03.035 PMC5394987 28283069

[61] Matsuda T Suzuki H Sugano Y Suzuki Y Yamanaka D Araki R. Effects of branched-chain amino acids on skeletal muscle, glycemic control, and neuropsychological performance in elderly persons with type 2 diabetes mellitus: an exploratory randomized controlled trial. Nutrients (2022) 14(19):3917. doi: 10.3390/nu14193917 36235570 PMC9572134

[62] Lee J Vijayakumar A White PJ Xu Y Ilkayeva O Lynch CJ. BCAA supplementation in mice with diet-induced obesity alters the metabolome without impairing glucose homeostasis. Endocrinology (2021) 162(7):bqab062. doi: 10.1210/endocr/bqab062 33765118 PMC8183497

[63] Newgard CB An J Bain JR Muehlbauer MJ Stevens RD Lien LF. A branched-chain amino acid-related metabolic signature that differentiates obese and lean humans and contributes to insulin resistance. Cell Metab (2009) 9(4):311–26. doi: 10.1016/j.cmet.2009.02.002 PMC3640280 19356713

[64] White PJ Lapworth AL An J Wang L McGarrah RW Stevens RD. Branched-chain amino acid restriction in Zucker-fatty rats improves muscle insulin sensitivity by enhancing efficiency of fatty acid oxidation and acyl-glycine export. Mol Metab (2016) 5(7):538–51. doi: 10.1016/j.molmet.2016.04.006 PMC4921791 27408778

[65] Cummings NE Williams EM Kasza I Konon EN Schaid MD Schmidt BA. Restoration of metabolic health by decreased consumption of branched-chain amino acids. J Physiol (2018) 596(4):623–45. doi: 10.1113/JP275075 PMC5813603 29266268

[66] Saha AK Xu XJ Lawson E Deoliveira R Brandon AE Kraegen EW. Downregulation of AMPK accompanies leucine- and glucose-induced increases in protein synthesis and insulin resistance in rat skeletal muscle. Diabetes (2010) 59(10):2426–34. doi: 10.2337/db09-1870 PMC3279521 20682696

[67] Schnuck JK Sunderland KL Gannon NP Kuennen MR Vaughan RA. Leucine stimulates PPARβ/δ-dependent mitochondrial biogenesis and oxidative metabolism with enhanced GLUT4 content and glucose uptake in myotubes. Biochimie (2016) 128-129:1–7. doi: 10.1016/j.biochi.2016.06.009 27345255

[68] Nishitani S Matsumura T Fujitani S Sonaka I Miura Y Yagasaki K. Leucine promotes glucose uptake in skeletal muscles of rats. Biochem Biophys Res Commun (2002) 299(5):693–6. doi: 10.1016/s0006-291x(02)02717-1 12470633

[69] Zhang Y Guo K LeBlanc RE Loh D Schwartz GJ Yu YH. Increasing dietary leucine intake reduces diet-induced obesity and improves glucose and cholesterol metabolism in mice via multimechanisms. Diabetes (2007) 56(6):1647–54. doi: 10.2337/db07-0123 17360978

[70] Karusheva Y Koessler T Strassburger K Markgraf D Mastrototaro L Jelenik T. Short-term dietary reduction of branched-chain amino acids reduces meal-induced insulin secretion and modifies microbiome composition in type 2 diabetes: a randomized controlled crossover trial. Am J Clin Nutr (2019) 110(5):1098–107. doi: 10.1093/ajcn/nqz191 PMC6821637 31667519

[71] Solerte SB Fioravanti M Locatelli E Bonacasa R Zamboni M Basso C. Improvement of blood glucose control and insulin sensitivity during a long-term (60 weeks) randomized study with amino acid dietary supplements in elderly subjects with type 2 diabetes mellitus. Am J Cardiol (2008) 101(11a):82e–8e. doi: 10.1016/j.amjcard.2008.03.006 18514633

[72] Nair KS Garrow JS Ford C Mahler RF Halliday D. Effect of poor diabetic control and obesity on whole body protein metabolism in man. Diabetologia (1983) 25(5):400–3. doi: 10.1007/BF00282518 6317503

[73] Louard RJ Barrett EJ Gelfand RA. Overnight branched-chain amino acid infusion causes sustained suppression of muscle proteolysis. Metabolism: Clin experimental (1995) 44(4):424–9. doi: 10.1016/0026-0495(95)90047-0 7723664

[74] Crown SB Marze N Antoniewicz MR. Catabolism of branched chain amino acids contributes significantly to synthesis of odd-chain and even-chain fatty acids in 3T3-L1 adipocytes. PloS One (2015) 10(12):e0145850. doi: 10.1371/journal.pone.0145850 26710334 PMC4692509

[75] Bishop CA Schulze MB Klaus S Weitkunat K. The branched-chain amino acids valine and leucine have differential effects on hepatic lipid metabolism. FASEB J (2020) 34(7):9727–39. doi: 10.1096/fj.202000195R 32506644

[76] Xiao F Huang Z Li H Yu J Wang C Chen S. Leucine deprivation increases hepatic insulin sensitivity via GCN2/mTOR/S6K1 and AMPK pathways. Diabetes (2011) 60(3):746–56. doi: 10.2337/db10-1246 PMC3046835 21282364

[77] Zhao H Zhang F Sun D Wang X Zhang X Zhang J. Branched-chain amino acids exacerbate obesity-related hepatic glucose and lipid metabolic disorders via attenuating akt2 signaling. Diabetes (2020) 69(6):1164–77. doi: 10.2337/db19-0920 32184272

[78] Mahendran Y Jonsson A Have CT Allin KH Witte DR Jørgensen ME. Genetic evidence of a causal effect of insulin resistance on branched-chain amino acid levels. Diabetologia (2017) 60(5):873–8. doi: 10.1007/s00125-017-4222-6 28184960

[79] Wang Q Holmes MV Davey Smith G Ala-Korpela M. Genetic support for a causal role of insulin resistance on circulating branched-chain amino acids and inflammation. Diabetes Care (2017) 40(12):1779–86. doi: 10.2337/dc17-1642 PMC5701741 29046328

[80] Vanweert F de Ligt M Hoeks J Hesselink MKC Schrauwen P Phielix E. Elevated plasma branched-chain amino acid levels correlate with type 2 diabetes-related metabolic disturbances. J Clin Endocrinol Metab (2021) 106(4):e1827–e36. doi: 10.1210/clinem/dgaa751 33079174

[81] Herman MA She P Peroni OD Lynch CJ Kahn BB. Adipose tissue branched chain amino acid (BCAA) metabolism modulates circulating BCAA levels. J Biol Chem (2010) 285(15):11348–56. doi: 10.1074/jbc.M109.075184 PMC2857013 20093359

[82] Suryawan A Hawes JW Harris RA Shimomura Y Jenkins AE Hutson SM. A molecular model of human branched-chain amino acid metabolism. Am J Clin Nutr (1998) 68(1):72–81. doi: 10.1093/ajcn/68.1.72 9665099

[83] Lackey DE Lynch CJ Olson KC Mostaedi R Ali M Smith WH. Regulation of adipose branched-chain amino acid catabolism enzyme expression and cross-adipose amino acid flux in human obesity. Am J Physiol Endocrinol Metab (2013) 304(11):E1175–87. doi: 10.1152/ajpendo.00630.2012 PMC3680678 23512805

[84] Burrill JS Long EK Reilly B Deng Y Armitage IM Scherer PE. Inflammation and ER stress regulate branched-chain amino acid uptake and metabolism in adipocytes. Mol Endocrinol (Baltimore Md) (2015) 29(3):411–20. doi: 10.1210/me.2014-1275 PMC4347289 25635940

[85] Li T Zhang Z Kolwicz SC Jr. Abell L Roe ND Kim M. Defective branched-chain amino acid catabolism disrupts glucose metabolism and sensitizes the heart to ischemia-reperfusion injury. Cell Metab (2017) 25(2):374–85. doi: 10.1016/j.cmet.2016.11.005 PMC5301464 28178567

[86] Cifarelli V Beeman SC Smith GI Yoshino J Morozov D Beals JW. Decreased adipose tissue oxygenation associates with insulin resistance in individuals with obesity. J Clin Invest (2020) 130(12):6688–99. doi: 10.1172/JCI141828 PMC7685757 33164985

[87] She P Van Horn C Reid T Hutson SM Cooney RN Lynch CJ. Obesity-related elevations in plasma leucine are associated with alterations in enzymes involved in branched-chain amino acid metabolism. Am J Physiol Endocrinol Metab (2007) 293(6):E1552–63. doi: 10.1152/ajpendo.00134.2007 PMC2767201 17925455

[88] Lefort N Glancy B Bowen B Willis WT Bailowitz Z De Filippis EA. Increased reactive oxygen species production and lower abundance of complex I subunits and carnitine palmitoyltransferase 1B protein despite normal mitochondrial respiration in insulin-resistant human skeletal muscle. Diabetes (2010) 59(10):2444–52. doi: 10.2337/db10-0174 PMC3279558 20682693

[89] Vanweert F Neinast M Tapia EE van de Weijer T Hoeks J Schrauwen-Hinderling VB. A randomized placebo-controlled clinical trial for pharmacological activation of BCAA catabolism in patients with type 2 diabetes. Nat Commun (2022) 13(1):3508. doi: 10.1038/s41467-022-31249-9 35717342 PMC9206682

[90] Zsuga J Török J Magyar MT Valikovics A Gesztelyi R Lenkei A. Dimethylarginines at the crossroad of insulin resistance and atherosclerosis. Metabolism: Clin experimental (2007) 56(3):394–9. doi: 10.1016/j.metabol.2006.10.023 17292729

[91] Schutte AE Schutte R Huisman HW van Rooyen JM Fourie CM Malan L. Dimethylarginines: their vascular and metabolic roles in Africans and Caucasians. Eur J endocrinol (2010) 162(3):525–33. doi: 10.1530/EJE-09-0865 19996198

[92] DiNicolantonio JJ McCarty MF OKeefe JH. Role of dietary histidine in the prevention of obesity and metabolic syndrome. Open heart (2018) 5(2):e000676. doi: 10.1136/openhrt-2017-000676 30018771 PMC6045700

[93] Fikri AM Smyth R Kumar V Al-Abadla Z Abusnana S Munday MR. Pre-diagnostic biomarkers of type 2 diabetes identified in the UAE’s obese national population using targeted metabolomics. Sci Rep (2020) 10(1):17616. doi: 10.1038/s41598-020-73384-7 33077739 PMC7572402

[94] Oxenkrug GF. Metabolic syndrome, age-associated neuroendocrine disorders, and dysregulation of tryptophan-kynurenine metabolism. Ann New York Acad Sci (2010) 1199:1–14. doi: 10.1111/j.1749-6632.2009.05356.x 20633104

[95] Scarale MG Mastroianno M Prehn C Copetti M Salvemini L Adamski J. Circulating metabolites associate with and improve the prediction of all-cause mortality in type 2 diabetes. Diabetes (2022) 71(6):1363–70. doi: 10.2337/db22-0095 35358315

[96] Rebnord EW Strand E Midttun Ø Svingen GFT Christensen MHE Ueland PM. The kynurenine:tryptophan ratio as a predictor of incident type 2 diabetes mellitus in individuals with coronary artery disease. Diabetologia (2017) 60(9):1712–21. doi: 10.1007/s00125-017-4329-9 PMC5552838 28612106

[97] Short KR Chadwick JQ Teague AM Tullier MA Wolbert L Coleman C. Effect of obesity and exercise training on plasma amino acids and amino metabolites in american Indian adolescents. J Clin Endocrinol Metab (2019) 104(8):3249–61. doi: 10.1210/jc.2018-02698 PMC6584131 31216576

[98] Wang TJ Ngo D Psychogios N Dejam A Larson MG Vasan RS. 2-Aminoadipic acid is a biomarker for diabetes risk. J Clin Invest (2013) 123(10):4309–17. doi: 10.1172/JCI64801 PMC3784523 24091325

[99] Lee HJ Jang HB Kim WH Park KJ Kim KY Park SI. 2-Aminoadipic acid (2-AAA) as a potential biomarker for insulin resistance in childhood obesity. Sci Rep (2019) 9(1):13610. doi: 10.1038/s41598-019-49578-z 31541119 PMC6754510

[100] Shi M Wang C Mei H Temprosa M Florez JC Tripputi M. Genetic architecture of plasma alpha-aminoadipic acid reveals a relationship with high-density lipoprotein cholesterol. J Am Heart Assoc (2022) 11(11):e024388. doi: 10.1161/JAHA.121.024388 35621206 PMC9238724

[101] Saremi A Howell S Schwenke DC Bahn G Beisswenger PJ Reaven PD. Advanced glycation end products, oxidation products, and the extent of atherosclerosis during the VA diabetes trial and follow-up study. Diabetes Care (2017) 40(4):591–8. doi: 10.2337/dc16-1875 PMC5360279 28148544

[102] Rhee EP Cheng S Larson MG Walford GA Lewis GD McCabe E. Lipid profiling identifies a triacylglycerol signature of insulin resistance and improves diabetes prediction in humans. J Clin Invest (2011) 121(4):1402–11. doi: 10.1172/JCI44442 PMC3069773 21403394

[103] Lu J Lam SM Wan Q Shi L Huo Y Chen L. High-coverage targeted lipidomics reveals novel serum lipid predictors and lipid pathway dysregulation antecedent to type 2 diabetes onset in normoglycemic chinese adults. Diabetes Care (2019) 42(11):2117–26. doi: 10.2337/dc19-0100 31455687

[104] Fabbrini E Sullivan S Klein S. Obesity and nonalcoholic fatty liver disease: biochemical, metabolic, and clinical implications. Hepatol (Baltimore Md) (2010) 51(2):679–89. doi: 10.1002/hep.23280 PMC3575093 20041406

[105] Salgin B Ong KK Thankamony A Emmett P Wareham NJ Dunger DB. Higher fasting plasma free fatty acid levels are associated with lower insulin secretion in children and adults and a higher incidence of type 2 diabetes. J Clin Endocrinol Metab (2012) 97(9):3302–9. doi: 10.1210/jc.2012-1428 22740706

[106] Ni Y Zhao L Yu H Ma X Bao Y Rajani C. Circulating unsaturated fatty acids delineate the metabolic status of obese individuals. EBioMedicine (2015) 2(10):1513–22. doi: 10.1016/j.ebiom.2015.09.004 PMC4634820 26629547

[107] Forouhi NG Imamura F Sharp SJ Koulman A Schulze MB Zheng J. Association of Plasma Phospholipid n-3 and n-6 Polyunsaturated Fatty Acids with Type 2 Diabetes: The EPIC-InterAct Case-Cohort Study. PloS Med (2016) 13(7):e1002094. doi: 10.1371/journal.pmed.1002094 27434045 PMC4951144

[108] Mahendran Y Cederberg H Vangipurapu J Kangas AJ Soininen P Kuusisto J. Glycerol and fatty acids in serum predict the development of hyperglycemia and type 2 diabetes in Finnish men. Diabetes Care (2013) 36(11):3732–8. doi: 10.2337/dc13-0800 PMC3816902 24026559

[109] Forouhi NG Koulman A Sharp SJ Imamura F Kröger J Schulze MB. Differences in the prospective association between individual plasma phospholipid saturated fatty acids and incident type 2 diabetes: the EPIC-InterAct case-cohort study. Lancet Diabetes endocrinol (2014) 2(10):810–8. doi: 10.1016/S2213-8587(14)70146-9 PMC4196248 25107467

[110] Sun L Liang L Gao X Zhang H Yao P Hu Y. Early prediction of developing type 2 diabetes by plasma acylcarnitines: A population-based study. Diabetes Care (2016) 39(9):1563–70. doi: 10.2337/dc16-0232 27388475

[111] Harris WS Luo J Pottala JV Margolis KL Espeland MA Robinson JG. Red blood cell fatty acids and incident diabetes mellitus in the women’s health initiative memory study. PloS One (2016) 11(2):e0147894. doi: 10.1371/journal.pone.0147894 26881936 PMC4755935

[112] Razquin C Toledo E Clish CB Ruiz-Canela M Dennis C Corella D. Plasma lipidomic profiling and risk of type 2 diabetes in the PREDIMED trial. Diabetes Care (2018) 41(12):2617–24. doi: 10.2337/dc18-0840 PMC6245212 30327364

[113] Lankinen MA Stančáková A Uusitupa M Ågren J Pihlajamäki J Kuusisto J. Plasma fatty acids as predictors of glycaemia and type 2 diabetes. Diabetologia (2015) 58(11):2533–44. doi: 10.1007/s00125-015-3730-5 26277381

[114] Lemaitre RN Fretts AM Sitlani CM Biggs ML Mukamal K King IB. Plasma phospholipid very-long-chain saturated fatty acids and incident diabetes in older adults: the Cardiovascular Health Study. Am J Clin Nutr (2015) 101(5):1047–54. doi: 10.3945/ajcn.114.101857 PMC4409688 25787996

[115] Guasch-Ferré M Ruiz-Canela M Li J Zheng Y Bulló M Wang DD. Plasma acylcarnitines and risk of type 2 diabetes in a mediterranean population at high cardiovascular risk. J Clin Endocrinol Metab (2019) 104(5):1508–19. doi: 10.1210/jc.2018-01000 PMC6435097 30423132

[116] Huang T Bhulaidok S Cai Z Xu T Xu F Wahlqvist ML. Plasma phospholipids n-3 polyunsaturated fatty acid is associated with metabolic syndrome. Mol Nutr Food Res (2010) 54(11):1628–35. doi: 10.1002/mnfr.201000025 20540149

[117] Fretts AM Imamura F Marklund M Micha R Wu JHY Murphy RA. Associations of circulating very-long-chain saturated fatty acids and incident type 2 diabetes: a pooled analysis of prospective cohort studies. Am J Clin Nutr (2019) 109(4):1216–23. doi: 10.1093/ajcn/nqz005 PMC6500926 30982858

[118] Fryk E Olausson J Mossberg K Strindberg L Schmelz M Brogren H. Hyperinsulinemia and insulin resistance in the obese may develop as part of a homeostatic response to elevated free fatty acids: A mechanistic case-control and a population-based cohort study. EBioMedicine (2021) 65:103264. doi: 10.1016/j.ebiom.2021.103264 33712379 PMC7992078

[119] Randle PJ Garland PB Hales CN Newsholme EA. The glucose fatty-acid cycle. Its role in insulin sensitivity and the metabolic disturbances of diabetes mellitus. Lancet (London England) (1963) 1(7285):785–9. doi: 10.1016/s0140-6736(63)91500-9 13990765

[120] Wanner C Tonelli M. KDIGO Clinical Practice Guideline for Lipid Management in CKD: summary of recommendation statements and clinical approach to the patient. Kidney Int (2014) 85(6):1303–9. doi: 10.1038/ki.2014.31 24552851

[121] Grant PJ Cosentino F. The 2019 ESC Guidelines on diabetes, pre-diabetes, and cardiovascular diseases developed in collaboration with the EASD: New features and the ‘Ten Commandments’ of the 2019 Guidelines are discussed by Professor Peter J. Grant and Professor Francesco Cosentino, the Task Force chairmen. Eur Heart J (2019) 40(39):3215–7. doi: 10.1093/eurheartj/ehz687 31608951

[122] Inoguchi T Li P Umeda F Yu HY Kakimoto M Imamura M. High glucose level and free fatty acid stimulate reactive oxygen species production through protein kinase C–dependent activation of NAD(P)H oxidase in cultured vascular cells. Diabetes (2000) 49(11):1939–45. doi: 10.2337/diabetes.49.11.1939 11078463

[123] Xu YN Wang Z Zhang SK Xu JR Pan ZX Wei X. Low-grade elevation of palmitate and lipopolysaccharide synergistically induced β-cell damage via inhibition of neutral ceramidase. Mol Cell endocrinol (2022) 539:111473. doi: 10.1016/j.mce.2021.111473 34610358

[124] Luo F Feng Y Ma H Liu C Chen G Wei X. Neutral ceramidase activity inhibition is involved in palmitate-induced apoptosis in INS-1 cells. Endocrine J (2017) 64(8):767–76. doi: 10.1507/endocrj.EJ16-0512 28674283

[125] Lee SM Choi SE Lee JH Lee JJ Jung IR Lee SJ. Involvement of the TLR4 (Toll-like receptor4) signaling pathway in palmitate-induced INS-1 beta cell death. Mol Cell Biochem (2011) 354(1-2):207–17. doi: 10.1007/s11010-011-0820-7 21503675

[126] Shi H Kokoeva MV Inouye K Tzameli I Yin H Flier JS. TLR4 links innate immunity and fatty acid-induced insulin resistance. J Clin Invest (2006) 116(11):3015–25. doi: 10.1172/JCI28898 PMC1616196 17053832

[127] Hirosumi J Tuncman G Chang L Görgün CZ Uysal KT Maeda K. A central role for JNK in obesity and insulin resistance. Nature (2002) 420(6913):333–6. doi: 10.1038/nature01137 12447443

[128] Yuan M Konstantopoulos N Lee J Hansen L Li ZW Karin M. Reversal of obesity- and diet-induced insulin resistance with salicylates or targeted disruption of Ikkbeta. Sci (New York NY) (2001) 293(5535):1673–7. doi: 10.1126/science.1061620 11533494

[129] Weatherill AR Lee JY Zhao L Lemay DG Youn HS Hwang DH. Saturated and polyunsaturated fatty acids reciprocally modulate dendritic cell functions mediated through TLR4. J Immunol (Baltimore Md: 1950) (2005) 174(9):5390–7. doi: 10.4049/jimmunol.174.9.5390 15843537

[130] Imamura F Micha R Wu JH de Oliveira Otto MC Otite FO Abioye AI. Effects of saturated fat, polyunsaturated fat, monounsaturated fat, and carbohydrate on glucose-insulin homeostasis: A systematic review and meta-analysis of randomized controlled feeding trials. PloS Med (2016) 13(7):e1002087. doi: 10.1371/journal.pmed.1002087 27434027 PMC4951141

[131] Drogan D Dunn WB Lin W Buijsse B Schulze MB Langenberg C. Untargeted metabolic profiling identifies altered serum metabolites of type 2 diabetes mellitus in a prospective, nested case control study. Clin Chem (2015) 61(3):487–97. doi: 10.1373/clinchem.2014.228965 25524438

[132] Fall T Salihovic S Brandmaier S Nowak C Ganna A Gustafsson S. Non-targeted metabolomics combined with genetic analyses identifies bile acid synthesis and phospholipid metabolism as being associated with incident type 2 diabetes. Diabetologia (2016) 59(10):2114–24. doi: 10.1007/s00125-016-4041-1 PMC5451119 27406814

[133] Floegel A Stefan N Yu Z Mühlenbruch K Drogan D Joost HG. Identification of serum metabolites associated with risk of type 2 diabetes using a targeted metabolomic approach. Diabetes (2013) 62(2):639–48. doi: 10.2337/db12-0495 PMC3554384 23043162

[134] Zhao J Zhu Y Hyun N Zeng D Uppal K Tran VT. Novel metabolic markers for the risk of diabetes development in American Indians. Diabetes Care (2015) 38(2):220–7. doi: 10.2337/dc14-2033 PMC4302260 25468946

[135] Park JE Lim HR Kim JW Shin KH. Metabolite changes in risk of type 2 diabetes mellitus in cohort studies: A systematic review and meta-analysis. Diabetes Res Clin practice (2018) 140:216–27. doi: 10.1016/j.diabres.2018.03.045 29626587

[136] Huynh K Barlow CK Jayawardana KS Weir JM Mellett NA Cinel M. High-throughput plasma lipidomics: detailed mapping of the associations with cardiometabolic risk factors. Cell Chem Biol (2019) 26(1):71–84.e4. doi: 10.1016/j.chembiol.2018.10.008 30415965

[137] Lemaitre RN Yu C Hoofnagle A Hari N Jensen PN Fretts AM. Circulating sphingolipids, insulin, HOMA-IR, and HOMA-B: the strong heart family study. Diabetes (2018) 67(8):1663–72. doi: 10.2337/db17-1449 PMC6054436 29588286

[138] Xia JY Holland WL Kusminski CM Sun K Sharma AX Pearson MJ. Targeted induction of ceramide degradation leads to improved systemic metabolism and reduced hepatic steatosis. Cell Metab (2015) 22(2):266–78. doi: 10.1016/j.cmet.2015.06.007 PMC4527941 26190650

[139] Raichur S Wang ST Chan PW Li Y Ching J Chaurasia B. CerS2 haploinsufficiency inhibits β-oxidation and confers susceptibility to diet-induced steatohepatitis and insulin resistance. Cell Metab (2014) 20(4):687–95. doi: 10.1016/j.cmet.2014.09.015 25295789

[140] Kolak M Westerbacka J Velagapudi VR Wågsäter D Yetukuri L Makkonen J. Adipose tissue inflammation and increased ceramide content characterize subjects with high liver fat content independent of obesity. Diabetes (2007) 56(8):1960–8. doi: 10.2337/db07-0111 17620421

[141] Coen PM Hames KC Leachman EM DeLany JP Ritov VB Menshikova EV. Reduced skeletal muscle oxidative capacity and elevated ceramide but not diacylglycerol content in severe obesity. Obes (Silver Spring Md) (2013) 21(11):2362–71. doi: 10.1002/oby.20381 PMC4136513 23512750

[142] Adams JM 2nd Pratipanawatr T Berria R Wang E DeFronzo RA Sullards MC. Ceramide content is increased in skeletal muscle from obese insulin-resistant humans. Diabetes (2004) 53(1):25–31. doi: 10.2337/diabetes.53.1.25 14693694

[143] Obeid LM Linardic CM Karolak LA Hannun YA. Programmed cell death induced by ceramide. Sci (New York NY) (1993) 259(5102):1769–71. doi: 10.1126/science.8456305 8456305

[144] Holland WL Brozinick JT Wang LP Hawkins ED Sargent KM Liu Y. Inhibition of ceramide synthesis ameliorates glucocorticoid-, saturated-fat-, and obesity-induced insulin resistance. Cell Metab (2007) 5(3):167–79. doi: 10.1016/j.cmet.2007.01.002 17339025

[145] Chaurasia B Kaddai VA Lancaster GI Henstridge DC Sriram S Galam DL. Adipocyte ceramides regulate subcutaneous adipose browning, inflammation, and metabolism. Cell Metab (2016) 24(6):820–34. doi: 10.1016/j.cmet.2016.10.002 27818258

[146] Adams SH Hoppel CL Lok KH Zhao L Wong SW Minkler PE. Plasma acylcarnitine profiles suggest incomplete long-chain fatty acid beta-oxidation and altered tricarboxylic acid cycle activity in type 2 diabetic African-American women. J Nutr (2009) 139(6):1073–81. doi: 10.3945/jn.108.103754 PMC2714383 19369366

[147] Kimura I Ozawa K Inoue D Imamura T Kimura K Maeda T. The gut microbiota suppresses insulin-mediated fat accumulation via the short-chain fatty acid receptor GPR43. Nat Commun (2013) 4:1829. doi: 10.1038/ncomms2852 23652017 PMC3674247

[148] Bjursell M Admyre T Göransson M Marley AE Smith DM Oscarsson J. Improved glucose control and reduced body fat mass in free fatty acid receptor 2-deficient mice fed a high-fat diet. Am J Physiol Endocrinol Metab (2011) 300(1):E211–220. doi: 10.1152/ajpendo.00229.2010 20959533

[149] Han JH Kim IS Jung SH Lee SG Son HY Myung CS. The effects of propionate and valerate on insulin responsiveness for glucose uptake in 3T3-L1 adipocytes and C2C12 myotubes via G protein-coupled receptor 41. PloS One (2014) 9(4):e95268. doi: 10.1371/journal.pone.0095268 24748202 PMC3991595

[150] Fujiwara K Maekawa F Yada T. Oleic acid interacts with GPR40 to induce Ca2+ signaling in rat islet beta-cells: mediation by PLC and L-type Ca2+ channel and link to insulin release. Am J Physiol Endocrinol Metab (2005) 289(4):E670–677. doi: 10.1152/ajpendo.00035.2005 15914509

[151] Nolan CJ Madiraju MS Delghingaro-Augusto V Peyot ML Prentki M. Fatty acid signaling in the beta-cell and insulin secretion. Diabetes (2006) 55 Suppl 2:S16–23. doi: 10.2337/db06-s003 17130640

[152] Chen Y Ren Q Zhou Z Deng L Hu L Zhang L. HWL-088, a new potent free fatty acid receptor 1 (FFAR1) agonist, improves glucolipid metabolism and acts additively with metformin in ob/ob diabetic mice. Br J Pharmacol (2020) 177(10):2286–302. doi: 10.1111/bph.14980 PMC7174891 31971610

[153] Li Z Liu C Zhou Z Hu L Deng L Ren Q. A novel FFA1 agonist, CPU025, improves glucose-lipid metabolism and alleviates fatty liver in obese-diabetic (ob/ob) mice. Pharmacol Res (2020) 153:104679. doi: 10.1016/j.phrs.2020.104679 32014571

[154] Akerfeldt MC Howes J Chan JY Stevens VA Boubenna N McGuire HM. Cytokine-induced beta-cell death is independent of endoplasmic reticulum stress signaling. Diabetes (2008) 57(11):3034–44. doi: 10.2337/db07-1802 PMC2570400 18591394

[155] Lin N Chen H Zhang H Wan X Su Q. Mitochondrial reactive oxygen species (ROS) inhibition ameliorates palmitate-induced INS-1 beta cell death. Endocrine (2012) 42(1):107–17. doi: 10.1007/s12020-012-9633-z 22350662

[156] Wu J Sun P Zhang X Liu H Jiang H Zhu W. Inhibition of GPR40 protects MIN6 β cells from palmitate-induced ER stress and apoptosis. J Cell Biochem (2012) 113(4):1152–8. doi: 10.1002/jcb.23450 22275065

[157] Kristinsson H Bergsten P Sargsyan E. Free fatty acid receptor 1 (FFAR1/GPR40) signaling affects insulin secretion by enhancing mitochondrial respiration during palmitate exposure. Biochim Biophys Acta (2015) 1853(12):3248–57. doi: 10.1016/j.bbamcr.2015.09.022 26408932

[158] Yamashima T. Dual effects of the non-esterified fatty acid receptor ‘GPR40’ for human health. Prog Lipid Res (2015) 58:40–50. doi: 10.1016/j.plipres.2015.01.002 25615413

[159] Marafie SK Al-Shawaf EM Abubaker J Arefanian H. Palmitic acid-induced lipotoxicity promotes a novel interplay between Akt-mTOR, IRS-1, and FFAR1 signaling in pancreatic β-cells. Biol Res (2019) 52(1):44. doi: 10.1186/s40659-019-0253-4 31426858 PMC6699284

[160] Finucane OM Lyons CL Murphy AM Reynolds CM Klinger R Healy NP. Monounsaturated fatty acid-enriched high-fat diets impede adipose NLRP3 inflammasome-mediated IL-1β secretion and insulin resistance despite obesity. Diabetes (2015) 64(6):2116–28. doi: 10.2337/db14-1098 25626736

[161] Chan KL Pillon NJ Sivaloganathan DM Costford SR Liu Z Théret M. Palmitoleate reverses high fat-induced proinflammatory macrophage polarization via AMP-activated protein kinase (AMPK). J Biol Chem (2015) 290(27):16979–88. doi: 10.1074/jbc.M115.646992 PMC4505442 25987561

[162] Oh DY Talukdar S Bae EJ Imamura T Morinaga H Fan W. GPR120 is an omega-3 fatty acid receptor mediating potent anti-inflammatory and insulin-sensitizing effects. Cell (2010) 142(5):687–98. doi: 10.1016/j.cell.2010.07.041 PMC2956412 20813258

[163] Souza CO Teixeira AA Lima EA Batatinha HA Gomes LM Carvalho-Silva M. Palmitoleic acid (n-7) attenuates the immunometabolic disturbances caused by a high-fat diet independently of PPARα. Mediators inflammation (2014) 2014:582197. doi: 10.1155/2014/582197 PMC4131426 25147439

[164] Im DS. Functions of omega-3 fatty acids and FFA4 (GPR120) in macrophages. Eur J Pharmacol (2016) 785:36–43. doi: 10.1016/j.ejphar.2015.03.094 25987421

[165] Hirasawa A Tsumaya K Awaji T Katsuma S Adachi T Yamada M. Free fatty acids regulate gut incretin glucagon-like peptide-1 secretion through GPR120. Nat Med (2005) 11(1):90–4. doi: 10.1038/nm1168 15619630

[166] Freitas RDS Campos MM. Understanding the appetite modulation pathways: The role of the FFA1 and FFA4 receptors. Biochem Pharmacol (2021) 186:114503. doi: 10.1016/j.bcp.2021.114503 33711286

[167] Landaas S. The formation of 2-hydroxybutyric acid in experimental animals. Clinica chimica acta; Int J Clin Chem (1975) 58(1):23–32. doi: 10.1016/0009-8981(75)90481-7 164303

[168] Sarswat PK Mishra YK Free ML. Fabrication and response of alpha-hydroxybutyrate sensors for rapid assessment of cardiometabolic disease risk. Biosensors bioelectronics (2017) 89(Pt 1):334–42. doi: 10.1016/j.bios.2016.07.019 27453438

[169] Gall WE Beebe K Lawton KA Adam KP Mitchell MW Nakhle PJ. alpha-hydroxybutyrate is an early biomarker of insulin resistance and glucose intolerance in a nondiabetic population. PloS One (2010) 5(5):e10883. doi: 10.1371/journal.pone.0010883 20526369 PMC2878333

[170] Varvel SA Pottala JV Thiselton DL Caffrey R Dall T Sasinowski M. Serum α-hydroxybutyrate (α-HB) predicts elevated 1 h glucose levels and early-phase β-cell dysfunction during OGTT. BMJ Open Diabetes Res Care (2014) 2(1):e000038. doi: 10.1136/bmjdrc-2014-000038 PMC4212560 25452875

[171] Nilsen MS Jersin R Ulvik A Madsen A McCann A Svensson PA. 3-hydroxyisobutyrate, A strong marker of insulin resistance in type 2 diabetes and obesity that modulates white and brown adipocyte metabolism. Diabetes (2020) 69(9):1903–16. doi: 10.2337/db19-1174 PMC7968520 32586980

[172] Molinaro A Bel Lassen P Henricsson M Wu H Adriouch S Belda E. Imidazole propionate is increased in diabetes and associated with dietary patterns and altered microbial ecology. Nat Commun (2020) 11(1):5881. doi: 10.1038/s41467-020-19589-w 33208748 PMC7676231

[173] Koh A Molinaro A Ståhlman M Khan MT Schmidt C Mannerås-Holm L. Microbially Produced Imidazole Propionate Impairs Insulin Signaling through mTORC1. Cell (2018) 175(4):947–61.e17. doi: 10.1016/j.cell.2018.09.055 30401435

[174] van Son J Serlie MJ Ståhlman M Bäckhed F Nieuwdorp M Aron-Wisnewsky J. Plasma imidazole propionate is positively correlated with blood pressure in overweight and obese humans. Nutrients (2021) 13(8):2706. doi: 10.3390/nu13082706 34444866 PMC8399073

[175] Prentice KJ Luu L Allister EM Liu Y Jun LS Sloop KW. The furan fatty acid metabolite CMPF is elevated in diabetes and induces β cell dysfunction. Cell Metab (2014) 19(4):653–66. doi: 10.1016/j.cmet.2014.03.008 24703697

[176] Yi J Jin H Zhang R Zhang S Chen P Yu X. Increased serum 3-carboxy-4-methyl-5-propyl-2-furanpropanoic acid (CMPF) levels are associated with glucose metabolism in Chinese pregnant women. J endocrinological Invest (2018) 41(6):663–70. doi: 10.1007/s40618-017-0789-5 PMC5951875 29151239

[177] Zhang S Chen P Jin H Yi J Xie X Yang M. Circulating 3-carboxy-4-methyl-5-propyl-2-furanpropanoic acid (CMPF) levels are associated with hyperglycemia and β cell dysfunction in a Chinese population. Sci Rep (2017) 7(1):3114. doi: 10.1038/s41598-017-03271-1 28596534 PMC5465180

[178] Zheng JS Lin M Imamura F Cai W Wang L Feng JP. Serum metabolomics profiles in response to n-3 fatty acids in Chinese patients with type 2 diabetes: a double-blind randomized controlled trial. Sci Rep (2016) 6:29522. doi: 10.1038/srep29522 27404516 PMC4941578

[179] Liu G Gibson RA Callahan D Guo XF Li D Sinclair AJ. Pure omega 3 polyunsaturated fatty acids (EPA, DPA or DHA) are associated with increased plasma levels of 3-carboxy-4-methyl-5-propyl-2-furanpropanoic acid (CMPF) in a short-term study in women. Food Funct (2020) 11(3):2058–66. doi: 10.1039/c9fo02440a 32142084

[180] Lankinen MA Hanhineva K Kolehmainen M Lehtonen M Auriola S Mykkänen H. CMPF does not associate with impaired glucose metabolism in individuals with features of metabolic syndrome. PloS One (2015) 10(4):e0124379. doi: 10.1371/journal.pone.0124379 25874636 PMC4398480

[181] Haeusler RA Astiarraga B Camastra S Accili D Ferrannini E. Human insulin resistance is associated with increased plasma levels of 12α-hydroxylated bile acids. Diabetes (2013) 62(12):4184–91. doi: 10.2337/db13-0639 PMC3837033 23884887

[182] Chávez-Talavera O Wargny M Pichelin M Descat A Vallez E Kouach M. Bile acids associate with glucose metabolism, but do not predict conversion from impaired fasting glucose to diabetes. Metabolism: Clin experimental (2020) 103:154042. doi: 10.1016/j.metabol.2019.154042 31785259

[183] Lee SG Lee YH Choi E Cho Y Kim JH. Fasting serum bile acids concentration is associated with insulin resistance independently of diabetes status. Clin Chem Lab Med (2019) 57(8):1218–28. doi: 10.1515/cclm-2018-0741 30964746

[184] Jørgensen NB Dirksen C Bojsen-Møller KN Kristiansen VB Wulff BS Rainteau D. Improvements in glucose metabolism early after gastric bypass surgery are not explained by increases in total bile acids and fibroblast growth factor 19 concentrations. J Clin Endocrinol Metab (2015) 100(3):E396–406. doi: 10.1210/jc.2014-1658 25536209

[185] Bishay RH Tonks KT George J Samocha-Bonet D Meyerowitz-Katz G Chisholm DJ. Plasma bile acids more closely align with insulin resistance, visceral and hepatic adiposity than total adiposity. J Clin Endocrinol Metab (2021) 106(3):e1131–e9. doi: 10.1210/clinem/dgaa940 33347566

[186] Safai N Suvitaival T Ali A Spégel P Al-Majdoub M Carstensen B. Effect of metformin on plasma metabolite profile in the Copenhagen Insulin and Metformin Therapy (CIMT) trial. Diabetic medicine: J Br Diabetic Assoc (2018) 35(7):944–53. doi: 10.1111/dme.13636 29633349

[187] Preiss D Rankin N Welsh P Holman RR Kangas AJ Soininen P. Effect of metformin therapy on circulating amino acids in a randomized trial: the CAMERA study. Diabetic medicine: J Br Diabetic Assoc (2016) 33(11):1569–74. doi: 10.1111/dme.13097 26887663

[188] Zhang Y Hu C Hong J Zeng J Lai S Lv A. Lipid profiling reveals different therapeutic effects of metformin and glipizide in patients with type 2 diabetes and coronary artery disease. Diabetes Care (2014) 37(10):2804–12. doi: 10.2337/dc14-0090 25011952

[189] Green CJ Marjot T Walsby-Tickle J Charlton C Cornfield T Westcott F. Metformin maintains intrahepatic triglyceride content through increased hepatic *de novo* lipogenesis. Eur J endocrinol (2022) 186(3):367–77. doi: 10.1530/EJE-21-0850 PMC8859923 35038311

[190] Huo T Cai S Lu X Sha Y Yu M Li F. Metabonomic study of biochemical changes in the serum of type 2 diabetes mellitus patients after the treatment of metformin hydrochloride. J Pharm Biomed analysis (2009) 49(4):976–82. doi: 10.1016/j.jpba.2009.01.008 19249171

[191] van der Meer RW Rijzewijk LJ de Jong HW Lamb HJ Lubberink M Romijn JA. Pioglitazone improves cardiac function and alters myocardial substrate metabolism without affecting cardiac triglyceride accumulation and high-energy phosphate metabolism in patients with well-controlled type 2 diabetes mellitus. Circulation (2009) 119(15):2069–77. doi: 10.1161/CIRCULATIONAHA.108.803916 19349323

[192] Jonker JT Wang Y de Haan W Diamant M Rijzewijk LJ van der Meer RW. Pioglitazone decreases plasma cholesteryl ester transfer protein mass, associated with a decrease in hepatic triglyceride content, in patients with type 2 diabetes. Diabetes Care (2010) 33(7):1625–8. doi: 10.2337/dc09-1935 PMC2890371 20150294

[193] Goldberg RB Kendall DM Deeg MA Buse JB Zagar AJ Pinaire JA. A comparison of lipid and glycemic effects of pioglitazone and rosiglitazone in patients with type 2 diabetes and dyslipidemia. Diabetes Care (2005) 28(7):1547–54. doi: 10.2337/diacare.28.7.1547 15983299

[194] Tahara N Yamagishi S-I Mizoguchi M Tahara A Imaizumi T. Pioglitazone decreases asymmetric dimethylarginine levels in patients with impaired glucose tolerance or type 2 diabetes. Rejuvenation Res (2013) (No.5):344–51. doi: 10.1089/rej.2013.1434 23777507

[195] Angelidi AM Kokkinos A Sanoudou D Connelly MA Alexandrou A Mingrone G. Early metabolomic, lipid and lipoprotein changes in response to medical and surgical therapeutic approaches to obesity. Metabolism: Clin experimental (2023) 138:155346. doi: 10.1016/j.metabol.2022.155346 36375643

[196] Peradze N Farr OM Perakakis N Lázaro I Sala-Vila A Mantzoros CS. Short-term treatment with high dose liraglutide improves lipid and lipoprotein profile and changes hormonal mediators of lipid metabolism in obese patients with no overt type 2 diabetes mellitus: a randomized, placebo-controlled, cross-over, double-blind clinical trial. Cardiovasc diabetol (2019) 18(1):141. doi: 10.1186/s12933-019-0945-7 31672146 PMC6823961

[197] Koska J Lopez L D’Souza K Osredkar T Deer J Kurtz J. Effect of liraglutide on dietary lipid-induced insulin resistance in humans. Diabetes Obes Metab (2018) 20(1):69–76. doi: 10.1111/dom.13037 28605158

[198] Zobel EH Wretlind A Ripa RS Rotbain Curovic V von Scholten BJ Suvitaival T. Ceramides and phospholipids are downregulated with liraglutide treatment: results from the LiraFlame randomized controlled trial. BMJ Open Diabetes Res Care (2021) 9(1):e002395. doi: 10.1136/bmjdrc-2021-002395 PMC8451300 34518158

[199] Rizzo M Chandalia M Patti AM Di Bartolo V Rizvi AA Montalto G. Liraglutide decreases carotid intima-media thickness in patients with type 2 diabetes: 8-month prospective pilot study. Cardiovasc Diabetol (2014) 13:49. doi: 10.1186/1475-2840-13-49 24559258 PMC3948058

[200] Jendle J Hyötyläinen T Orešič M Nyström T. Pharmacometabolomic profiles in type 2 diabetic subjects treated with liraglutide or glimepiride. Cardiovasc diabetol (2021) 20(1):237. doi: 10.1186/s12933-021-01431-2 34920733 PMC8684205

[201] Rodgers M Migdal AL Rodríguez TG Chen ZZ Nath AK Gerszten RE. Weight loss outcomes among early high responders to exenatide treatment: A randomized, placebo controlled study in overweight and obese women. Front endocrinol (2021) 12:742873. doi: 10.3389/fendo.2021.742873 PMC8635796 34867786

[202] Chaudhuri A Ghanim H Vora M Sia CL Korzeniewski K Dhindsa S. Exenatide exerts a potent antiinflammatory effect. J Clin Endocrinol Metab (2012) 97(1):198–207. doi: 10.1210/jc.2011-1508 22013105 PMC3251936

[203] Gerstein HC Lee SF Paré G Bethel MA Colhoun HM Hoover A. Biomarker changes associated with both dulaglutide and cardiovascular events in the REWIND randomized controlled trial: A nested case-control *post hoc* analysis. Diabetes Care (2023) 46(5):1046–51. doi: 10.2337/dc22-2397 36897834

[204] Pirro V Roth KD Lin Y Willency JA Milligan PL Wilson JM. Effects of tirzepatide, a dual GIP and GLP-1 RA, on lipid and metabolite profiles in subjects with type 2 diabetes. J Clin Endocrinol Metab (2022) 107(2):363–78. doi: 10.1210/clinem/dgab722 34608929

[205] Cakirca M Karatoprak C Zorlu M Kiskac M Kanat M Cikrikcioglu MA. Effect of vildagliptin add-on treatment to metformin on plasma asymmetric dimethylarginine in type 2 diabetes mellitus patients. Drug design Dev Ther (2014) 8:239–43. doi: 10.2147/DDDT.S52545 PMC3931658 24627624

[206] Boschmann M Engeli S Dobberstein K Budziarek P Strauss A Boehnke J. Dipeptidyl-peptidase-IV inhibition augments postprandial lipid mobilization and oxidation in type 2 diabetic patients. J Clin Endocrinol Metab (2009) 94(3):846–52. doi: 10.1210/jc.2008-1400 19088168

[207] Monami M Lamanna C Desideri CM Mannucci E. DPP-4 inhibitors and lipids: systematic review and meta-analysis. Adv Ther (2012) 29(1):14–25. doi: 10.1007/s12325-011-0088-z 22215383

[208] Irving BA Carter RE Soop M Weymiller A Syed H Karakelides H. Effect of insulin sensitizer therapy on amino acids and their metabolites. Metabolism: Clin experimental (2015) 64(6):720–8. doi: 10.1016/j.metabol.2015.01.008 PMC4525767 25733201

[209] Sathyanarayana P Jogi M Muthupillai R Krishnamurthy R Samson SL Bajaj M. Effects of combined exenatide and pioglitazone therapy on hepatic fat content in type 2 diabetes. Obes (Silver Spring Md) (2011) 19(12):2310–5. doi: 10.1038/oby.2011.152 21660077

